# Comparative Analysis of Circulating Noncoding RNAs Versus Protein Biomarkers in the Detection of Myocardial Injury

**DOI:** 10.1161/CIRCRESAHA.119.314937

**Published:** 2019-06-04

**Authors:** Christian Schulte, Temo Barwari, Abhishek Joshi, Konstantinos Theofilatos, Anna Zampetaki, Javier Barallobre-Barreiro, Bhawana Singh, Nils A. Sörensen, Johannes T. Neumann, Tanja Zeller, Dirk Westermann, Stefan Blankenberg, Michael Marber, Christoph Liebetrau, Manuel Mayr

**Affiliations:** 1 From the King’s British Heart Foundation Centre, King’s College London, United Kingdom (C.S., T.B., A.J., K.T., A.Z., J.B.-B., B.S., M. Mayr); 2 Bart’s Heart Centre, St. Bartholomew's Hospital, West Smithfield, London (A.J.); 3 Department of General and Interventional Cardiology, University Heart Centre Hamburg Eppendorf, Germany (C.S., N.A.S., J.T.N., T.Z., D.W., S.B.); 4 German Centre of Cardiovascular Research (DZHK), Partner Site Hamburg, Luebeck, Kiel, Germany (C.S., N.A.S., J.T.N., T.Z., D.W., S.B.); 5 King’s British Heart Foundation Centre, King’s College London, Guy’s and St Thomas’ Hospitals, United Kingdom (M. Marber); 6 Department of Cardiology, Kerckhoff Heart and Thorax Center, Bad Nauheim, Germany and German Centre of Cardiovascular Research (DZHK), Partner Site Rhine-Main, Bad Nauheim, Germany (C.L.).

**Keywords:** biomarkers, cardiac myosin-binding protein C, microRNAs, myocardial infarction, RNA, long noncoding, troponin

## Abstract

**Rationale:**

Noncoding RNAs (ncRNAs), including microRNAs (miRNAs), circular RNAs (circRNAs), and long noncoding RNAs (lncRNAs), are proposed novel biomarkers of myocardial injury. Their release kinetics have not been explored without confounding by heparin nor has their relationship to myocardial protein biomarkers.

**Objective:**

To compare ncRNA types in heparinase-treated samples with established and emerging protein biomarkers for myocardial injury.

**Methods and Results:**

Screening of 158 circRNAs and 21 lncRNAs in human cardiac tissue identified 12 circRNAs and 11 lncRNAs as potential biomarkers with cardiac origin. Eleven miRNAs were included. At low spike-in concentrations of myocardial tissue, significantly higher regression coefficients were observed across ncRNA types compared with cardiac troponins and cMyBP-C (cardiac myosin-binding protein C). Heparinase treatment of serial plasma and serum samples of patients undergoing transcoronary ablation of septal hypertrophy removed spurious correlations between miRNAs in non-heparinase-treated samples. After transcoronary ablation of septal hypertrophy, muscle-enriched miRNAs (miR-1 and miR-133a) showed a steeper and earlier increase than cardiac-enriched miRNAs (miR-499 and miR-208b). Putative cardiac lncRNAs, including LIPCAR (long intergenic noncoding RNA predicting cardiac remodeling and survival), did not rise, refuting a predominant cardiac origin. Cardiac circRNAs remained largely undetectable. In a validation cohort of acute myocardial infarction, receiver operating characteristic curve analysis revealed noninferiority of cardiac-enriched miRNAs, but miRNAs failed to identify cases presenting with low troponin values. cMyBP-C was validated as a biomarker with highly sensitive properties, and the combination of muscle-enriched miRNAs with high-sensitive cardiac troponin T and cMyBP-C returned the highest area under the curve values.

**Conclusions:**

In a comparative assessment of ncRNAs and protein biomarkers for myocardial injury, cMyBP-C showed properties as the most sensitive cardiac biomarker while miRNAs emerged as promising candidates to integrate ncRNAs with protein biomarkers. Sensitivity of current miRNA detection is inferior to cardiac proteins but a multibiomarker combination of muscle-enriched miRNAs with cMyBP-C and cardiac troponins could open a new path of integrating complementary characteristics of different biomarker types.

## Abstract 2

Supplemental Digital Content is available in the text.

In its most recent and fourth definition of myocardial infarction (MI), the European Society of Cardiology has refined approaches to classify and differentiate MI.^1^ While higher sensitivity troponin assays have improved the identification of low-risk patients suitable for immediate discharge, detecting, and treating minor cardiac damage may fail to result in better clinical outcomes.^2^ There is still a need for biomarkers that facilitate early rule-out/rule-in of clinically relevant MI. Using proteomics, we discovered that cMyBP-C (cardiac myosin-binding protein C) is released earlier upon myocardial ischemia than cardiac troponins^3^ and may contribute to a better rule-out/rule-in classification of MI.^4^

**Editorial, see p 341**

**Meet the First Author, see p 260**

Recently, noncoding RNAs (ncRNAs) have been implicated as biomarkers of MI. MicroRNAs (miRNAs), long noncoding RNAs (lncRNAs), and circular RNAs (circRNAs) are among the ncRNAs present in the circulation. Plasma and serum levels of muscle- and cardiac-enriched miRNAs increase markedly after MI.^5,6^ Besides miRNAs, lncRNAs, and circRNAs have attracted interest as potential biomarkers in cardiovascular disease. Levels of the long intergenic ncRNA predicting cardiac remodeling (LIPCAR) were reported to predict adverse cardiac remodeling and death after MI.^7^ However, circRNAs as a different ncRNA type are less susceptible to RNase activity and may offer tissue specificity with >15 000 circRNAs being present in the human heart.^8,9^

Heparin, an anticoagulant commonly administered in the clinical setting of MI, is a major confounding factor for measurements of ncRNAs by real-time polymerase chain reaction (qPCR). Few studies on the release of ncRNAs after MI used heparinase treatment to overcome this confounding effect by heparin, a prerequisite for comparative analysis of ncRNA and protein biomarkers. Moreover, while circulating levels of muscle- and cardiac-enriched miRNAs have been shown to correlate to troponins after MI, differences in the release of ncRNAs and novel protein biomarkers such as cMyBP-C have not been compared in the clinically most relevant setting of MI patients presenting early with low troponin values.

The objective of this study was to use heparinase treatment to establish the release kinetics of 3 different types of ncRNAs (miRNAs, lncRNAs, and circRNAs) in serial samples from patients undergoing transcoronary ablation of septal hypertrophy (TASH) as well as in patients with acute MI presenting with a wide range of hs-cTn (high-sensitive cardiac troponin) levels in the BACC study (Biomarkers in Acute Cardiac Care; n>2500 patients). The performance of ncRNAs was compared with hs-cTn and cMyBP-C as established and novel protein biomarkers of cardiac injury, respectively.

## Methods

The authors declare that all supporting data are available within the article and its Online Data Supplement. Larger data sets, such as array data, are available from the corresponding author on reasonable request.

### RNA Extraction

Total RNA was extracted using the miRNeasy Mini kit (Qiagen, Hilden, Germany) according to the manufacturer’s recommendations, with some modifications. In brief, 100 µL of serum or plasma were combined with 694.75 µL of Qiazol lysis reagent, 4 µL of diluted synthetic *Caenorhabditis elegans* miR-39 (*Cel-miR-39-3p*) spike-in and 1.25 µL carrier RNA from bacteriophage MS2 (Roche). After a brief incubation at room temperature, 140 µL of chloroform was added and the solution was mixed vigorously. Samples were then centrifuged at 13500×*g* for 15 minutes at 4°C. Two hundred eighty microliters of the upper (aqueous) phase were transferred to a new tube and mixed with 1.5 volumes (420 µL) of 100% ethanol and applied to columns and washed according to the manufacturer’s protocol. Total RNA was eluted in 35 µL of nuclease-free H_2_O by centrifugation at 8500×*g* for 1 minute at 4°C.

### Heparinase Treatment

#### ncRNA Analyses

Before reverse transcription, the extracted RNA was treated with heparinase 1 from Flavobacterium heparinum (Sigma) according to the following protocol: 5 µL of each sample were combined with 1.25 µL heparinase, 0.25 µL of RNase inhibitor (Ribo Lock 40 U/µL, Thermofisher) and 3.5 µL of heparinase buffer (pH 7.5) and thoroughly mixed, then incubated at 25°C for 3 hours. The samples were then immediately used for reverse transcription. For comparison, a buffer-only group was treated with heparinase buffer devoid of heparinase, which was incubated under the same conditions as the heparinase-treated samples. The untreated group received neither heparinase nor buffer, nor was it left for incubation, but instead was used for further reverse transcription together with the treated samples.

#### Proximity Extension Assay

cTnI was part of the organ damage panel offered by Olink (Uppsala, Sweden). Human plasma samples were treated by adding 0.1 U (concentration: 0.2 U/µL) of heparinase 1 per 1 µL of plasma. 0.5 µL of the heparinase solution was added per 1 µL of plasma. The mixture was then incubated for 1 hour at 30°C as previously described.^10^

### Reverse Transcription

For reverse transcription, 2 different platforms (1) for miRNAs (miRCURY LNA RT kit [Exiqon]) and (2) for lncRNAs and circRNAs (SuperScript VILO MasterMix [Invitrogen]) were used. For further details see Online Data Supplement.

### Real-Time PCR Assays

A list of primers used for qPCR detection and their sequence is provided in Online Table I. For further details see the Online Data Supplement.

### RNA Quantification

In the analyses of raw quantification cycle (Cq) data, any measurements beyond 35 cycles were considered undetectable. For details see the Online Data Supplement. In brief, the quantification for RNAs was performed as follows:

#### Analysis of miRNAs

In TASH samples as well as the MI cohort the delta-delta Cq method was used for relative quantification, using *Cel-miR-39-3p* as normalization control. Quantification results were calibrated against the median of 3 identical replicates consisting of equal volumes from all TASH or all MI samples, respectively. Relative quantification was performed with Microsoft Excel, version 15.32 for MacOS. In the myocardial tissue in vitro spike-in experiment normalization was also performed using *Cel-miR-39* spike-in. Calibration was performed using the median value of all samples per individual RNA assay to remove assay-related biases.

#### Analysis of circRNAs and lncRNAs

Calibration of circRNAs and lncRNAs in the TASH samples was performed against the median of all samples for each assay. In the myocardial tissue spike-in experiment, the same method was used. The relative quantity was calculated as described above for miRNAs.

### Myocardial Tissue Spike-In Experiment

To assess the detectability of RNAs with cardiac origin, different amounts of human heart tissue were spiked into human plasma. For details see Online Data Supplement and Online Figure I.

### Selection of ncRNAs

Two cardiac-enriched miRNAs (miR-208b-3p and miR-499a-5p), 2 muscle-enriched miRNAs (miR-1 and miR-133a-3p), and 7 additional miRNAs were included in the analyses based on their good detectability in human plasma as noncardiac/nonmuscle counterparts. For circRNAs, we performed a microarray-based screening in 4 pooled samples per time point of the TASH cohort (n=16 pooled samples; Arraystar Human Circular RNA Array, Arraystar INC, Rockville, MD). For details see Online Data Supplement. To complement this screening, a literature search was performed. circRNAs were selected from 4 deep sequencing data sets reporting >15 000 circRNAs, of which 158 circRNAs were RNase R-treated and validated by qPCR. These 158 circRNAs plus their linear transcripts were first tested in 12 human cardiac tissue samples. For circRNAs with more than one transcript from the same gene, the one with the best detection based on Cq values was chosen. Only circRNAs with Cq values of <25 cycles that were derived from cardiac and muscle-associated genes (n=12) were included. lncRNAs were selected based on microarray screening of 33 045 lncRNAs in human plasma.^7^ Of these, 768 lncRNAs showed differential plasma levels in patients developing heart failure after MI. Twenty-one lncRNAs with high signal intensity were validated by qPCR. As for circRNAs, only lncRNAs with Cq values <25 (n=11) were selected for further analyses. A graphical depiction of the selection process including references is shown in Online Figure II.

### Statistical Analyses

#### Tissue Spike-In Experiment

To enable comparisons of the relative expression values for ncRNAs and the absolute concentrations for protein biomarkers, relative quantities have been calculated for all molecules by dividing their values with the median quantity for each molecule. Subsequently, linear regression curves were calculated for all miRNAs, circRNAs, lncRNAs, and cardiac proteins to study their release kinetics by comparing the regression curves slopes. All *R*^2^ values were >0.9, therefore, the used linear model provides a good fit to the data. Next, the slopes of the regression curves of the 3 molecules with the highest scores were selected from each category; for miRNAs: miR-133a, miR-208b, and miR-499; for circRNAs: circALPK2, circMYBPC3, and circSLC8A1; for lncRNAs: lncDANCR, lncH19, and lncRNACOX2; and for proteins: hs-cTnT, hs-cTnI, and cMyBP-C. Mann-Whitney *U* tests^11^ were used to perform pairwise statistical comparisons between the slopes of proteins and ncRNAs.

#### TASH Cohort

To study the release kinetics of proteins and ncRNAs at 1 hour after TASH, absolute protein measurements and relative RNA measurements were both transformed into relative values on the same scale by dividing each value by the overall maximum value of the single biomarker across all time points. The data for each molecule and each patient were curve-fitted using linear regression and slopes of the curves were calculated. Mann-Whitney *U* tests were used to perform pairwise statistical comparisons between the slopes of the regression curves of protein and ncRNA molecules.

#### BACC Study

Analyses to study release kinetics after acute MI were performed analogous to the TASH cohort for the first hour after hospital presentation. Correlation analyses of biomarkers in the acute MI cohort were performed with Graph Pad Prism 7.0d for MacOS. Nonparametric Spearman correlation was used because none of the biomarkers were normally distributed. *P* values in the correlation analyses were 2-tailed, and approximate values were calculated.

#### Receiver Operating Characteristic Analyses

For training and testing regression models for predicting the time from onset of MI combining miRNAs and proteins, we used a hybrid of a heuristic algorithm and Support Vector Regression models (details are provided in the Online Data Supplement).

## Results

### Detectability of ncRNAs in Human Plasma

**Figure 1.**
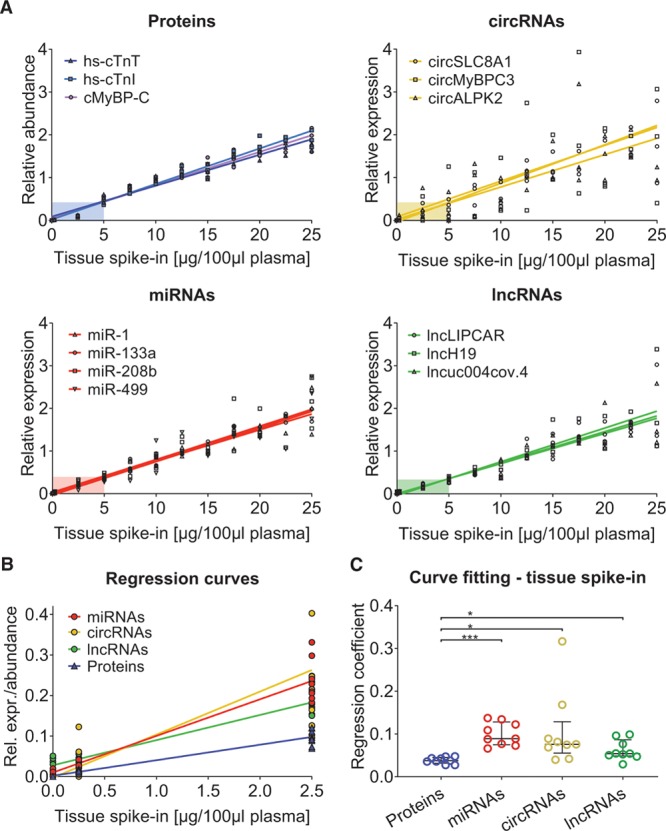
**Myocardial tissue spike-in.** **A**, Linear regression curves of each of the 3 noncoding RNA (ncRNA) classes with the highest coefficient of determination (*R*^2^) values. At low spike-in concentrations, the measured protein concentrations were markedly below the regression curve (colored boxes). **B**, Linear regression curves of all biomarker types, combining the single biomarkers from **A** per class. ncRNAs showed steeper regression curves compared with protein biomarkers. **C**, At low spike-in concentrations, significantly higher regression coefficients, indicating higher sensitivity, were observed for all ncRNA types compared with proteins. cMyBP-C indicates cardiac myosin-binding protein C; hs-cTnI, high-sensitive cardiac troponin I; and hs-cTnT, high-sensitive cardiac troponin T; and lncRNA, long noncoding RNA. ***P<0.0001; *P<0.0028 (Mann-Whitney *U* test).

To compare detectability of different ncRNAs human myocardial tissue was spiked into plasma of healthy volunteers at defined concentrations of 0.25 µg to 25µg/100 µL plasma (Online Figure I). Based on published data, we selected 158 circRNAs and 21 lncRNAs that were reported as abundant in human myocardium (for details see Methods section and Online Figure II). circRNAs associated with cardiac-specific proteins, such as cTnT, cTnI, and cMyBP-C, were among the least well-detectable circRNAs in plasma (data not shown). Muscle- (miR-1 and miR-133a) and cardiac-enriched miRNAs (miR-208b and miR-499) were chosen for comparison. These 4 miRNAs showed comparable regression curves to cardiac circRNAs (circSLC8A1, circMyBPC3, and circALPK2) and lncRNAs (lncLIPCAR and lncH19 lncuc004.cov4; Figure 1A, Online Figures III, IV, and V). Levels of other miRNAs remained unaltered on spiking human myocardial tissue into plasma (Online Figure VI). Next, ncRNA spike-in results were compared with measurements of established and novel cardiac protein biomarkers as previously described^12^ (Online Figure VII). While ncRNAs demonstrated a continuous, linear dose-response curve across all spike-in concentrations, measurements of cardiac proteins (hs-cTnT, hs-cTnI, and cMyBP-C) remained below their regression curve at low spike-in concentrations (0.25 µg and 2.5 µg/100 µL plasma; Figure 1A, colored boxes). At low spike-in concentrations, ncRNA regression curves were steeper compared with cardiac protein biomarkers (Figure 1B). Curve fitting analyses for low spike-in concentrations returned significantly higher regression coefficients for ncRNAs (Figure 1C, Mann-Whitney *U* test for comparison against cardiac protein biomarkers: miRNAs *P*<0.0001, fold-change 2.6; circRNAs *P*=0.0028, fold-change 2.8; and lncRNAs *P*=0.0028, fold-change 1.6).

### Confounding by Heparin in ncRNA Analysis

**Figure 2.**
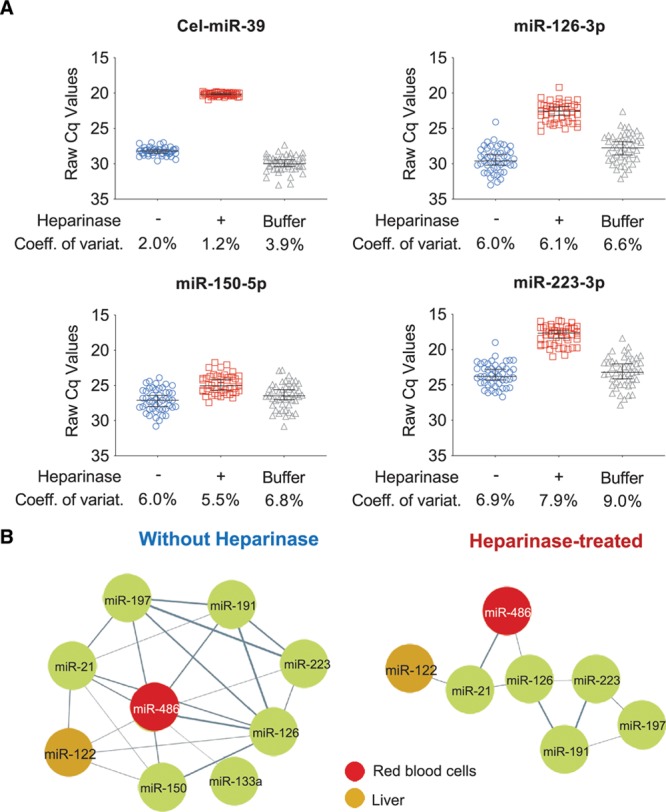
**Heparin effect on selected microRNAs (miRNAs) and results after heparinase treatment**. **A**, Human plasma samples were treated with heparin after blood was drawn, then miRNA expression was measured (blue). The measurements were repeated in the same samples after they were treated with heparinase (red) or with a buffer solution lacking heparinase (gray). **B**, Clustering networks of miRNAs in the transcoronary ablation of septal hypertrophy (TASH) cohort: before heparinase treatment the analyzed miRNAs showed a dense correlation network. Heparinase treatment resolved this clustering of miRNAs, removing spurious correlations between miRNAs in non-heparinase-treated TASH samples. The distinct cellular origin of miRNAs, ie, liver-specific miR-122 vs red blood cell-associated miR-486, became more readily apparent. Coeff. of variat. indicates coefficient of variation.

The derangements of ncRNA biomarker measurements after heparin administration can be addressed by heparinase treatment as demonstrated in 2 examples: First, human plasma was spiked with 10 IU of heparin per 1 mL plasma. Heparin reduced the detectability of the exogenous spike-in control *Cel-miR-39* and endogenous miRNAs resulting in elevated raw Cq values, which was reversed by heparinase treatment (Figure 2A). Second, we assessed samples from a cohort of patients undergoing TASH,^13^ where the exact time points of myocardial injury and heparin administration were known, and samples were obtained at baseline before myocardial injury. We evaluated plasma miRNA levels before and 1 hour, 8 hours, and 24 hours after induced myocardial injury in TASH patients (n=16). In non-heparinase-treated samples, we discovered a dense miRNA correlation network, which consists of spurious correlations between miRNAs independent of their cellular origin. This observation contradicts the well-known cell- and tissue-associated expression of miRNAs (Figure 2B). Notably, liver-specific miR-122 and red blood cell-enriched miR-486 appeared in the same cluster area. Heparinase treatment resolved the clustering of the network removing spurious correlations between miRNAs in non-heparinase-treated samples. Thus, the distinct cellular origins of plasma miRNAs became readily apparent: as visualized in Figure 2B, the clustering shows liver miR-122 and red blood cell miR-486 in separate cluster areas from previously reported platelet-enriched miRNAs (miR-126, miR-223, and miR-191).

### Release Kinetics of ncRNAs After TASH

**Figure 3.**
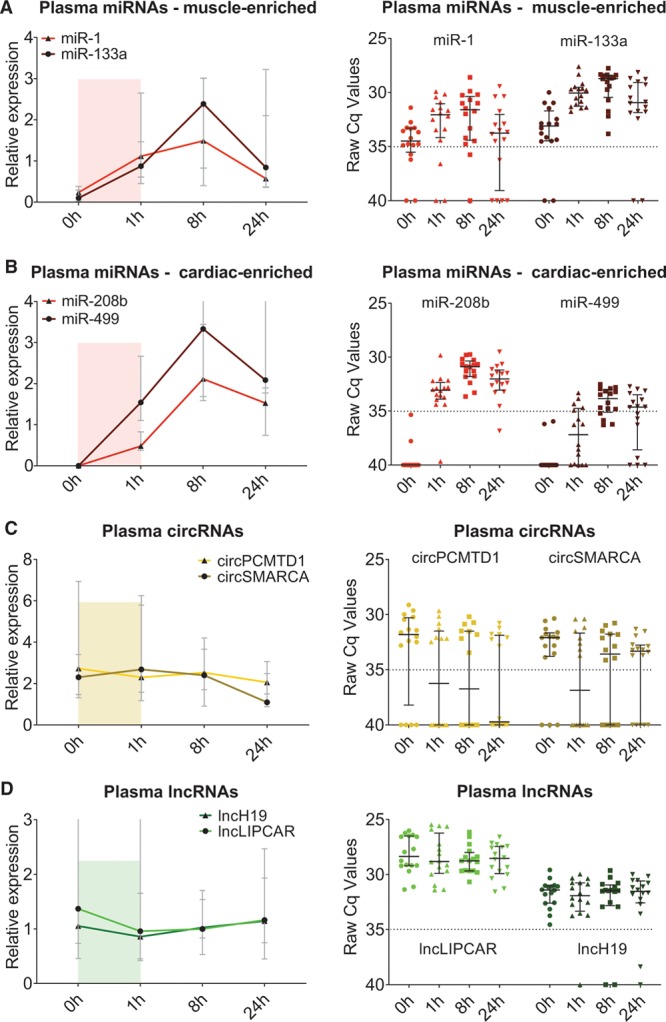
**Noncoding RNAs (ncRNAs) after transcoronary ablation of septal hypertrophy (TASH).** Relative plasma levels as well as raw Cq values for muscle-enriched (**A**) and cardiac-enriched (**B**) microRNAs (miRNAs) as well as circular RNAs (circRNAs) (**C**) and long noncoding RNA (lncRNAs) **(D)** after TASH for time points before (0 h) and after myocardial injury (1 h, 8 h, and 24 h). Dotted line indicates the detectability threshold; Cq values above 35 were considered as undetectable. Of particular interest is the time course of the first hour after TASH with respect to biomarker sensitivity (colored boxes). Depicted are median values, error bars indicate interquartile range.

To assess the release of ncRNAs after myocardial injury, serial samples were obtained from patients undergoing TASH.^14^ On heparinase treatment, the release of ncRNAs was compared with hs-cTnT and cMyBP-C at baseline, 1 hour, 8 hours, and 24 hours after induced myocardial injury. The clinical characteristics of the TASH patients were reported previously.^14^ Plasma and serum from 16 patients at 4 time points were used for comparative analyses of the release of muscle- (miR-1 and miR-133a; Figure 3A) and of cardiac-enriched miRNAs (miR-208b and miR-499; Figure 3B), circRNAs (circSMARCA and circPCMTDL; Figure 3C), and lncRNAs (lncLIPCAR and lncH19; Figure 3D). These circRNAs and lncRNAs were chosen for their best detectability in plasma and serum. Unlike muscle-enriched miRNAs, the 2 cardiac-enriched miRNAs were undetectable at baseline. MiR-208b became detectable at 1 hour after TASH. For miR-499, detectable levels were only reached at 8 hours after TASH. Neither mitochondrial lncRNA LIPCAR nor nucleus-derived lncRNA H19 changed after TASH (Figure 3D). Thus, LIPCAR and lncRNA H19 levels are not of cardiac origin. Unlike other ncRNA classes, cardiac circRNAs showed poor detectability at baseline and after TASH (Figure 3C), despite the fact that these circRNAs were readily detectable in cardiac tissue (Online Figure VIII). An additional circRNA microarray screening of 13 617 circRNAs performed at all 4 time points did not return any significantly dysregulated circRNAs after TASH (Online Figure IX).

### Comparison of Cardiac Protein Versus ncRNA Biomarkers in TASH

**Figure 4.**
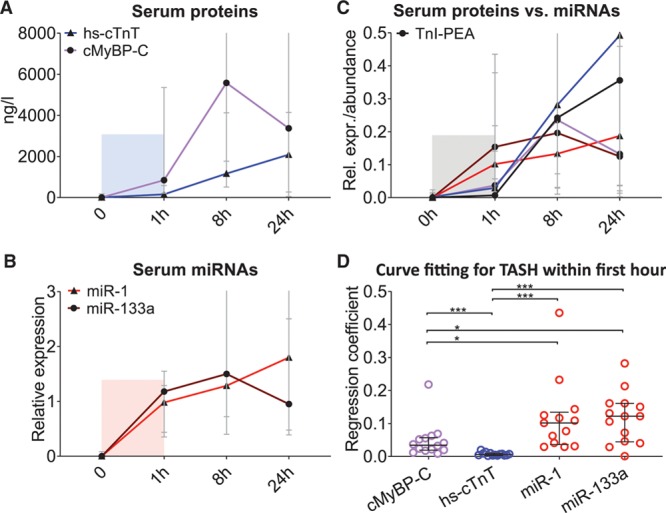
**Noncoding RNAs (ncRNAs) and protein biomarkers after transcoronary ablation of septal hypertrophy (TASH).** **A**, Levels of cardiac protein biomarkers in the TASH cohort. **B**, Levels of muscle-enriched miRNAs in the TASH cohort. **C**, Transformation of absolute protein quantification measures and relative miRNA quantification measures plus additional relative measures by proximity extension assays (PEA, Olink) to the same scale by dividing each value by the maximum value of each biomarker. **D**, Slope statistics on the relative expression of miRNAs after transformation according to panel B revealed significant differences in the regression coefficients between muscle-enriched miRNAs (miR-1 and miR-133a) and protein biomarkers for the first hour after TASH (time point 0 and 1h). ***P<0.0001; *P<0.01 (Mann-Whitney *U* test) relative to the maximum value of each biomarker. Panels **A**–**C** show median values, error bars indicate interquartile range. TnI-PEA denotes cardiac troponin I as measured by PEA.

Figure 4 depicts the time course of serum levels of cardiac protein biomarkers (hs-TnT and cMyBP-C; Figure 4A) and circulating muscle-enriched miRNAs after TASH (miR-1 and miR-133a; Figure 4B). As reported previously,^15^ cMyBP-C levels peaked before hs-TnT. Similarly, muscle-enriched miRNAs (miR-1 and miR-133a) peaked earlier than cardiac protein biomarkers. When the measurements of cardiac proteins and the 2 muscle-enriched miRNAs, miR-1 and miR-133a, were expressed as proportions of the maximum detected value (Figure 4C), cMyBP-C showed a significantly higher regression coefficient compared with hs-cTnT (*P*<0.0001, Mann-Whitney *U* test; Figure 4D). Similarly, higher regression coefficients were observed for muscle-enriched miRNAs compared with cardiac protein biomarkers within the first hour after induced myocardial injury (Mann-Whitney *U* test; hs-TnT versus miR-1 *P*<0.0001, hs-TnT versus miR-133a *P*<0.0001, cMyBP-C versus miR-1 *P*=0.0091, and cMyBP-C versus miR-133a *P*=0.0088; Figure 4D).

Circulating miRNAs and proteins were measured by qPCR and ELISA, respectively. To rule-out that the different assay methodology impacts on the observed release kinetics, cTnI was assessed using a proximity extension assay (PEA). The PEA combines dual antibody-based detection with qPCR-based quantification. While the PEA for cTnI was less sensitive compared with hs-cTnT, both assays revealed a similar temporal profile for the cTn release after TASH (Figure 4C). The PEA measurements of cTnI were not affected by heparin (Online Figure X) because of the minute amount of sample input required compared with miRNA measurements (1 µL of plasma for cTnI versus 100 µL for miRNAs).

### Comparison of miRNAs, cMyBP-C, and Troponins in Patients With Acute MI

**Figure 5.**
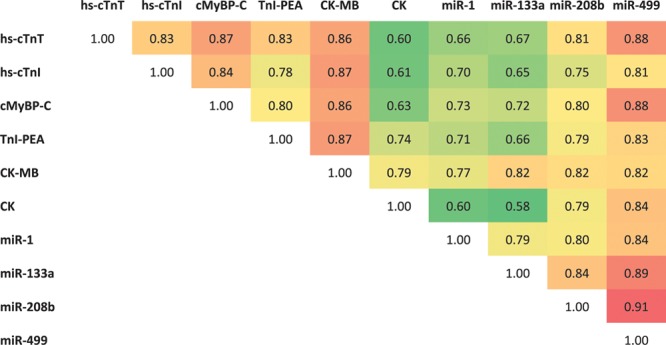
**Correlation of cardiac biomarkers in acute myocardial infarction (MI).** All analyzed biomarkers are highly correlated. Cardiac-enriched microRNAs (miRNAs) correlate better with hs-cTnT (high-sensitive cardiac troponin T) and among each other than muscle-enriched miRNAs. Depicted are regression coefficients; *P* for all combinations <0.0001. CK indicates creatine kinase; CK-MB, creatine kinase muscle/brain; cMyBP-C, cardiac myosin-binding protein C; hs-cTnI, high-sensitive cardiac troponin I; and TnI-PEA, cardiac troponin I as measured by a proximity extension assay (PEA, Olink).

To compare miRNA kinetics in patients with acute MI, we analyzed plasma samples from a carefully selected subcohort (n=83) of the BACC study,^16^ focusing on patients with initially low hs-cTnT levels which show a steep increase within the first hour after hospital presentation (Online Table II). Samples were taken on admission, 1 hour and 3 hours thereafter in 38 acute MI patients. 45 patients with noncardiac chest pain served as controls. The plasma levels of muscle- and cardiac-enriched miRNAs strongly correlated with concentrations of hs-cTnT, hs-cTnI, and cMyBP-C (Figure 5). Correlations were stronger in patients with ST-segment–elevation MI (STEMI, n=20) than in patients with non–ST-segment–elevation myocardial infarction (NSTEMI, n=18; Online Figure XI). As expected, given their cardiac enrichment, the highest correlation with hs-cTnT was observed for the 2 cardiac-enriched miRNAs, miR-208b, and miR-499 (*r*=0.81 and *r*=0.88, respectively, *P*<0.0001), which is as high a correlation level as between hs-cTnT and cMyBP-C and hs-cTnT and hs-cTnI (*r*=0.87 and *r*=0.83, respectively, *P*<0.0001). The correlation was substantially weaker for the 2 muscle-enriched miRNAs, miR-1, and miR-133a (*r*=0.67 for both, *P*<0.0001). Correlations of miRNAs with cMyBP-C were comparable with correlations of miRNAs with hs-cTnT.

**Figure 6.**
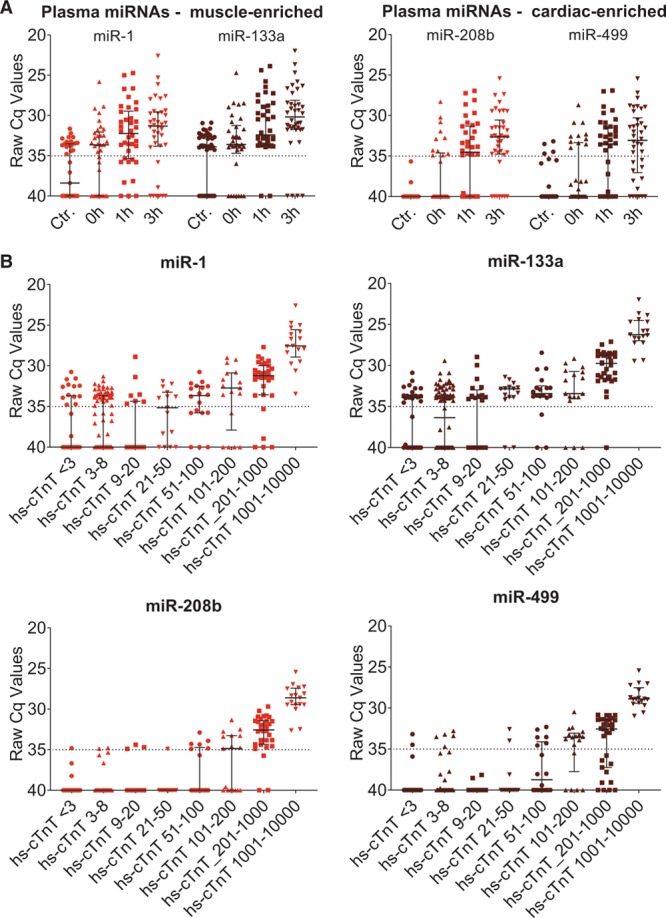
**MicroRNA (miRNA) raw expression data in the myocardial infarction (MI) cohort**. **A**, Raw Cq values of miRNAs for control patients (Ctr) and MI patients according to time of admission to hospital (0 h on presentation at hospital, 1 h and 3 h after presentation). At every time point, there were undetectable values (Cq>35) for each miRNA. **B**, miRNA raw Cq values corresponding to different ranges of hs-cTnT (high-sensitive cardiac troponin T) concentrations (ng/L). Only for hs-cTnT levels above 1000 ng/L all miRNAs showed 100% detectability. Black dotted line denotes lower limit of detection (Cq>35).

Because the diagnosis of acute MI was adjudicated based on hs-cTnT, hs-cTnI was measured.^16^ Data for hs-cTnI were comparable with hs-cTnT (Online Figure XIIA and XIIB). At time point 0 and 1 hour, one sample was below the lower limit of detection for hs-cTnT for each time point, while hs-cTnT was detectable in all MI patients at 3 hours. In contrast, all miRNAs showed numerous undetectable values in the MI group (Figure 6A). Again, this was more pronounced in NSTEMI patients than in STEMI patients (Online Figure XIII). Hs-cTnT was above the lower limit of detection in 85% of the control patients (n=45), while cMyBP-C levels were above the lower limit of detection in 100% of the measurements, including control patients (Online Figures XIIC and XIV).

Next, miRNA levels were reported for defined hs-cTnT groups (Figure 6B). Only at high hs-cTnT concentrations (>1000 ng/L), miRNAs were detectable in all MI patients. At low-positive hs-cTnT levels (comprising hs-cTnT levels between 21 and 50 ng/L), miR-1, miR-133a, miR-208b, and miR-499 were detectable in 47%, 87%, 7%, and 13% of patients, respectively. In patients with hs-cTnT concentrations below 10 ng/mL, miR-208b and miR-499 remained below the detection threshold (Cq of >35) whereas miR-1 and miR-133a were detectable in 39% and 64% of patients, respectively. To validate this finding, we included an additional group of 19 carefully selected MI patients with hs-cTnT levels of <1000 ng/L at all 3 time points (n=57 samples, Online Table III). In an attempt to maximize detectability, we doubled the input of RNA for the RT-qPCR reaction. The rise in miR-1, miR-133a, miR-208b, and miR-499, however, was mainly detectable at hs-cTnT levels >50 to 100 ng/L (Online Figure XV).

**Figure 7.**
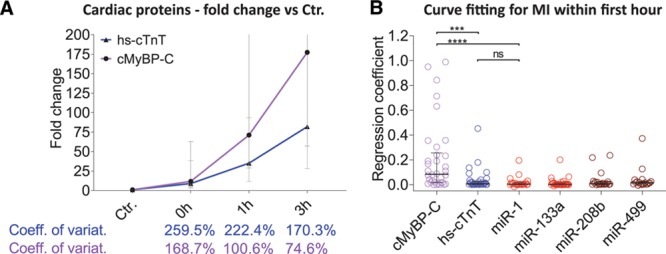
**Release kinetics of cardiac protein biomarkers and curve fitting in myocardial infarction (MI) cohort**. **A**, cMyBP-C (cardiac myosin-binding protein C) rose more quickly after onset of MI compared with hs-cTnT (high-sensitive cardiac troponin T) with still a smaller coefficient of variation (values depicted are median with interquartile range). **B**, Curve fitting analysis of the first hour after hospital presentation revealed significantly higher regression coefficients for cMyBP-C than all other biomarkers. ns indicates not significant (Mann-Whitney *U* test). ****P<0.0001; ***P=0.0002. Ctr indicates control patients; and miR; microRNA.

Analogous to the TASH results, miR-1 (44%) and miR-133a (63%) were also more readily detectable in the control group compared with miR-208b (0%) and miR-499 (10%; Online Figure XIV). cMyBP-C was detectable in all control and MI samples. Confirming the results from the TASH patients, in the MI cohort, cMyBP-C showed a steeper increase shortly after hospital presentation than hs-cTnT with yet smaller coefficients of variation at all time points in the MI cohort (Figure 7A). Curve fitting analysis revealed significantly higher regression coefficients for cMyBP-C than for hs-cTnT and muscle and cardiac miRNAs (Figure 7B).

### Comparison of Receiver Operating Characteristic Analyses Based on the TASH and MI Cohorts

**Figure 8.**
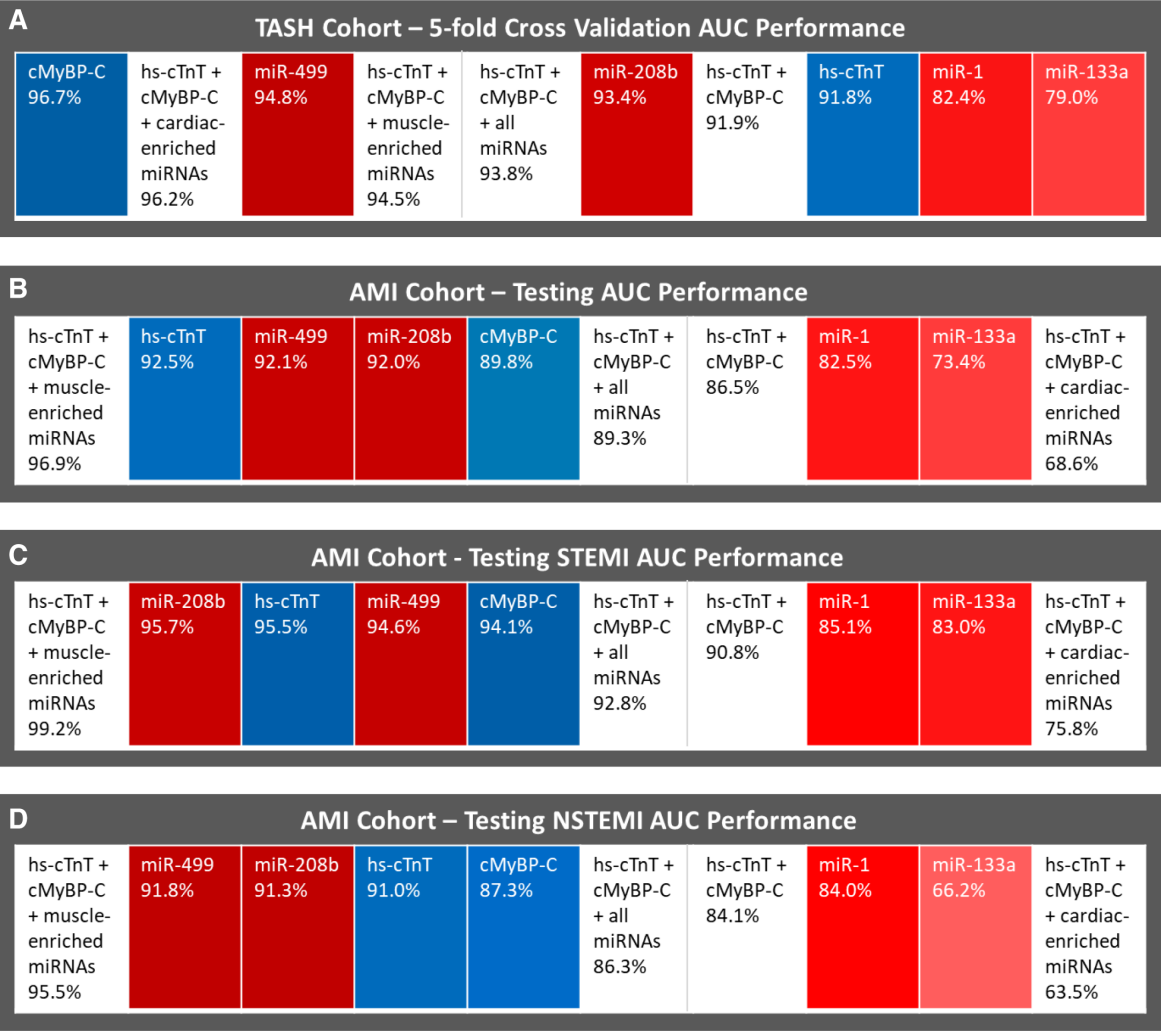
**Receiver operating characteristic (ROC) analysis comparing predictive power of protein and microRNA (miRNA) biomarkers**. **A**, Transcoronary ablation of septal hypertrophy (TASH) cohort. **B**, Myocardial infarction (MI) cohort with subanalysis for (**C**) ST-segment–elevation myocardial infarction (STEMI) patients. **D**, Non–ST-segment–elevation myocardial infarction (NSTEMI) patients. Blue color for proteins, red for miRNAs, white for combinations of 2 or more biomarkers. The biomarkers are ranked from left to right (highest to lowest area under the curve [AUC] value). The intensity of the blue and red color increases with increasing AUC values.

When comparing patients before the TASH procedure with any of the time points after (1 hour, 8 hours, and 24 hours), both miRNAs-208b and -499 showed a higher predictive value (area under the curve [AUC], 0.934 and 0.948, respectively) than hs-cTnT (0.918) for the detection of myocardial injury (Figure 8A, Online Figure XVI and Table IV). The combination of hs-cTnT with miR-208b or miR-499 improved AUC values to 0.943 and 0.957, respectively. A combination of both cardiac-enriched miRNAs offered no further improvement in the predictive value. AUC values for miR-1 and miR-133a were lower (0.824 and 0.790, respectively) despite their higher sensitivity. cMyBP-C was the cardiac biomarker with the highest predictive power (0.967).

Next, the MI cohort was used as an independent validation cohort for the predictive analytics from TASH. Thus, the most promising regression models of the different combinations of proteins and miRNAs were applied to the MI cohort. When comparing control patients with acute MI patients at any of the time points (0 hour, 1 hour, and 3 hours), both miRNAs-208b and -499 showed similar power (AUC values of 0.920 and 0.921, respectively) as hs-cTnT (0.925) for the prediction of MI. This result is comparable to the receiver operating characteristic (ROC) analyses in the TASH cohort. AUC values for miR-1 and miR-133a were lower (0.825 and 0.734, respectively) despite their higher sensitivity in plasma, demonstrating the lack of cardiac specificity of muscle-enriched miRNAs (Figure 8B, Online Figure XVII and Table V). The highest AUC value in TASH was observed for cMyBP-C (0.967), while in the MI cohort, the best performance in the ROC analysis was observed for the combination of hs-TnT, cMyBP-C, and muscle-enriched miRNAs (0.969).

Finally, cases of STEMI and NSTEMI (excluding NSTEMI type 2) were assessed separately to explore different causes of myocardial injury. The ROC analyses presented in Figure 8C and 8D indicate that the performance of the diagnostic models is better in STEMI patients. The performance in the NSTEMI group was inferior to both STEMI and all MI cases. Importantly, the ranking of biomarker performance was consistent in all 3 comparisons, independent of the overall performance of the model.

## Discussion

Thus far, most attention has focused on miRNAs although new classes of ncRNAs have been identified in the circulation.^17,18^ In addition to miRNAs, we assessed the potential of selected lncRNAs and circRNAs to serve as biomarkers of myocardial injury. To the best of our knowledge, no study has directly compared these classes of ncRNAs with cardiac protein biomarkers. Using heparinase-treated samples, we assessed the release of ncRNAs after TASH in a well-controlled context of cardiac injury and of miRNAs in the most relevant clinical setting of MI cases presenting with low initial troponin values.

### Heparinase Treatment to Overcome Confounding by Heparin

Heparin inhibits qPCR-based ncRNA quantification.^19^ Confounding by heparin is evidenced by decreased detectability, higher variation, or spurious correlations of ncRNA measurements. Apart from endogenous miRNAs, heparin predominantly affects the quantification of the exogenous *Cel-miR-39* spike-in control. As pointed out previously,^20^ the inter-sample deviation of *Cel-miR-39* measurements should be <1 cycle. However, within the first hour after administration of the heparin bolus, the detectability of *Cel-miR-39* decreases by approximately 3 cycles. This variability is related to the half-life of heparin in the circulation. Most publications assessing miRNAs in samples from patients with MI failed to address this issue (Online Table VI). If unnoticed, heparin-induced suppression of the *Cel-miR-39* normalization control results in artificially higher levels of endogenous miRNAs, especially within the first hour after heparin administration (Figure 2B). Heparinase treatment can overcome the confounding introduced by heparin in samples from MI patients.^21^ This is the first time that heparinase treatment was performed on a clinical MI cohort before ncRNA quantification. Our analyses of miRNAs in MI patients returned substantially higher correlation coefficients with cardiac troponins than previous publications that did not use heparinase.^21^

### NcRNAs and Protein Biomarkers in Myocardial Injury

Assays for cTnI and cTnT are the gold standard for detection of myocardial injury.^22,23^ The excellent sensitivity of these assays is the result of decades of optimization.^24,25^ Using proteomics, we have recently identified a new cardiac biomarker, cMyBP-C, which may allow for an earlier detection and better rule-in/rule-out of MI.^3,4^ In our assessment, cMyBP-C detected myocardial injury with a higher accuracy than hs-cTnT and hs-cTnI in the controlled TASH model. This finding is supported by a steeper rise within the first hour after TASH and in all time intervals (0–1 hour and 1–3 hours) in the MI cohort. cMyBP-C showed a higher detectability among control patients compared with hs-cTnT (100% versus 85%, respectively). However, ROC analysis in the validation cohort of MI revealed lower AUC for cMyBP-C compared with hs-cTnT (0.898 versus 0.925). This finding is most likely attributable to 2 factors: First, as opposed to TASH, the diagnosis of MI was adjudicated based on hs-cTnT; and second, the selection of the MI patients from the BACC cohort was determined by initially low and then steeply rising hs-cTnT levels. Importantly, cMyBP-C has been reported as more sensitive compared with cTn.^4^ This is supported by our finding that cMyBP-C shows a steeper rise in the first hour after onset of myocardial injury. The better detectability of cMyBP-C in controls also suggests that cMyBP-C might be a biomarker for cardiac disease in non-acute settings.

In addition to cardiac proteins, miRNAs offer a new opportunity for the detection of myocardial injury. The muscle-enriched miRNAs, miR-1 and miR-133a, have been implicated as markers for cardiac injury but are not specific for the heart. In contrast, miR-499 and miR-208a/b have higher cardiac specificity but are less abundant in heart and in plasma.^26^ By spiking plasma with human myocardial tissue, we demonstrated that qPCR assays for ncRNAs detect the presence of smaller amounts of myocardial tissue than cardiac proteins. The regression curves for ncRNAs compared with protein biomarkers indicated a potentially higher sensitivity of qPCR-based measurements of ncRNAs. In a tightly controlled clinical setting of induced myocardial injury after TASH, muscle- and cardiac-enriched miRNAs showed an earlier rise than hs-cTnT, which was similar to the release kinetics of cMyBP-C (Figure 3A and 3B and Figure 4A and 4B). To exclude that this difference derives from the mode of measurement, we also performed additional cTnI measurements with PEA, which combines antibody-based detection with qPCR-based quantification. Another important aspect for biomarker performance is the clearance of cardiac proteins and miRNAs from the circulation. Similar to cMyBP-C, miRNA levels peaked at 8 hours and declined or plateaued thereafter. In contrast, hs-cTnT concentrations were still rising at 24 hours after TASH. While single miRNAs failed to outperform cardiac protein biomarkers in detecting early MI, a multibiomarker combination of 2 muscle-enriched miRNAs with hs-cTnT and cMyBP-C returned the highest predictive power for the detection of MI in a subcohort of the BACC study. This was consistent across different causes of myocardial injury (STEMI and NSTEMI type 1). While the biomarker selection and their ranking did not change, the overall performance of the prediction model was largely dependent on infarct severity.

### Cardiac- and Muscle-Enriched miRNAs

Although cardiac and muscle-enriched miRNAs have been previously studied as biomarker candidates for myocardial injury, our findings in heparinase-treated samples highlight important aspects that have, to our knowledge, not been addressed so far. First, the muscle-enriched miRNAs, miR-1 and miR-133a, are more readily detectable at baseline, while miR-208 and -499 reach detectable levels only at higher corresponding hs-cTnT concentrations.^5^ However, cardiac-enriched miRNAs are more specific for myocardial injury. They correlate best with hs-cTnT and predict myocardial injury better in the TASH cohort and as good as hs-cTnT in the MI cohort. This is consistent with previous reports of miR-208 and miR-499 being elevated in plasma only in cases of MI or myocardial injury, while miR-1 and miR-133a can rise in different cardiac pathologies.^6,27^ In a ROC analysis of the TASH cohort, cardiac-enriched miRNAs returned higher AUC values than muscle-enriched miRNAs. Second, cardiac miRNA measurements in patients with MI reached the detection limit of Cq<35 cycles only at high hs-cTnT values (>50–100 ng/L). Thus, miRNAs failed to identify patients with MI that initially present with low or negative cTn values. A critical evaluation of publications is required as higher thresholds of detection may have been used in some studies and confounding by heparin was not taken into consideration (Online Table VI). Given their favorable release kinetics, this shortcoming of cardiac miRNA biomarkers might be attributed to the low miRNA yield from plasma, miRNA degradation after release into circulation and inadequate detection methods compared to high-sensitivity protein assays.

### LncRNAs

LncRNAs are a heterogeneous group of RNAs with >200 nucleotides in length.^28^ Unlike miRNAs, lncRNAs are mainly located within the nucleus or in mitochondria.^7,29^ Regardless, lncRNAs are readily detected in the circulation, suggesting some protection against RNase-mediated degradation similar to plasma miRNAs.^28^ LIPCAR was found to predict adverse cardiac remodeling and death in the aftermath of MI.^7^ LIPCAR has been proposed as a biomarker of cardiac disease. However, the cardiac origin of this lncRNA in plasma has not been confirmed. In our experiments, LIPCAR showed a comparable regression curve to cardiac miRNAs in the myocardial tissue spike-in and good detectability in plasma. LIPCAR levels, however, did not increase after TASH, refuting a cardiac origin. Because LIPCAR is of mitochondrial origin and ubiquitously expressed, its rise in plasma may be explained by a release of mitochondria from blood cells rather than cardiac injury.^30^

### CircRNAs

CircRNAs are expressed in a tissue- and developmental-specific manner.^9^ They can either emerge from exons or introns of pre-mRNA and are products of alternative splicing known as backsplicing.^8^ circRNAs have diverse functions^31,32^ and are tissue-specific.^33^ Sequencing data revealed the presence of >15 000 circRNAs in the human heart, some in high abundance.^9^ circRNAs have previously been implicated in MI-related apoptosis.^34^ The majority of circRNAs are located in the cytoplasm,^35^ which increases the probability of their early release upon tissue damage. circRNAs have been described as circulating biomarkers in the field of oncology.^36^ Our study is the first to assess circRNAs as biomarkers in acute MI. circRNAs in plasma showed poor detectability despite high abundance in cardiac tissue. Also, circRNAs did not show a rise in plasma after myocardial injury. While circRNAs are supposedly less prone to degradation compared with their linear transcripts,^37^ this may differ in the circulation where circRNAs have been described as having a short half-life.^37^ Thus, cardiac circRNAs were not well detectable in plasma and serum.

### Conclusions

In summary, heparinase treatment is essential when evaluating ncRNAs in clinical settings. Among ncRNAs, cardiac miRNAs remained the best predictor for the diagnosis of acute MI. In serial samples from TASH and acute MI patients, cardiac miRNAs showed comparable AUC values to hs-cTnT and the additional use of muscle-enriched miRNAs combined with hs-cTnT and cMyBP-C returned the highest AUC for MI, pointing out their potential in combined protein/ncRNA biomarker approaches. However, miRNA sensitivity proved to be well below hs-cTnT, arguing against their clinical application at the current stage of methodological advances. Thus, analyses in larger cohorts seem warranted once technological advances offer better sensitivity. Future miRNA assays also require faster, automated quantification if miRNAs were to be used in a clinical setting for complementing protein biomarkers. With regards to cardiac proteins, measurements of cMyBP-C could offer some of the benefits of miRNAs, as evidenced by an earlier rise and faster decline after myocardial injury and a better baseline detectability compared with cardiac troponins.

## Acknowledgments

We acknowledge Christian Cassel for technical assistance. We are grateful to Dr Tom Kaier for providing values of previously published cMyBP-C measurements. We thank Alina Goßling and Dr Francisco M. Ojeda for data handling of the BACC study.

## Sources of Funding

M. Mayr is a British Heart Foundation (BHF) Chair Holder (CH/16/3/32406) with BHF programme grant support (RG/16/14/32397); was awarded a BHF Special Project grant to participate in the ERA-CVD Transnational Grant “MacroERA: Noncoding RNAs in cardiac macrophages and their role in heart failure” and is part of the Marie Skłodowska-Curie Innovative Training Network TRAIN-HEART (http://train-heart.eu) as well as a network on “MicroRNA-based Therapeutic Strategies in Vascular Disease” funded by the Foundation Leducq. This work was supported by the National Institute of Health Research (NIHR) Biomedical Research Centre based at Guy’s and St Thomas’ NHS (National Health Service) Foundation Trust and King’s College London in partnership with King’s College Hospital. C. Schulte is the recipient of a research fellowship by the Deutsche Forschungsgemeinschaft (DFG; SCHU 2983/1-1 and SCHU 2983/1–2). T. Barwari was funded by a BHF Interdisciplinary PhD studentship. A. Joshi is a BHF Clinical Research Training Fellow (FS/16/32/32184). A. Zampetaki was an Intermediate Fellow of the BHF (FS/13/18/30207). T. Zeller is funded by the German Centre for Cardiovascular Research (DZHK; 81Z0710102) and supported by a European Research Area Network (ERA-Net; PREMED-CAD). The BACC study was supported by an unrestricted grant by Abbott Diagnostics. It was further funded in part by the German Centre of Cardiovascular Research (DZHK e.V.). N.A. Sörensen and J.T. Neumann were supported by grants from the German Heart Foundation/German Foundation of Heart Research. J.T. Neumann was supported by the Else Kröner Fresenius Stiftung.

## Disclosures

M. Mayr and M. Marber are named inventors on a licensed patent held by King’s College London for the detection of cMyBP-C as a biomarker of myocardial injury (EP2430453B1, US8546089). M. Mayr filed and licensed patent applications on miRNAs as biomarkers (EP15193448.6, EP2776580 B1, DE112013006129T5, GB2524692A, EP2576826 B, and JP2013-513740). J.T. Neumann received honoraria from Siemens and Abbott Diagnostics. D. Westermann reports personal fees from Bayer, Boehringer-Ingelheim, Berlin Chemie, Astra Zeneca, Biotronik and Novartis. S. Blankenberg received honoraria from Abbott Diagnostics, Siemens, Thermo Fisher, and Roche Diagnostics and is a consultant for Thermo Fisher. The other authors report no conflicts.

## Supplementary Material

## Novelty and Significance

### What Is Known?

- High-sensitivity cardiac troponins are the gold standard biomarkers for myocardial infarction (MI).
- Noncoding RNAs (ncRNAs) have been reported as candidate biomarkers for MI.
- No study has compared different classes of ncRNAs with cardiac protein biomarkers.
