# Supplementary material for: Comparative Analysis of Circulating Noncoding RNAs Versus Protein Biomarkers in the Detection of Myocardial Injury
Source: Circ Res. 2019 Jun 4;125(3):328–40. doi: 10.1161/CIRCRESAHA.119.314937 (PMC6641471; doi:10.1161/CIRCRESAHA.119.314937)
Supplement: Supplementary file 1 [file res-125-328-s001.pdf]

SUPPLEMENTAL MATERIAL

A Comparative Analysis of Circulating Non-Coding RNAs versus Protein Biomarkers in the Detection of Myocardial Injury

Christian Schulte MD[a,b,c], Temo Barwari MD, PhD[a], Abhishek Joshi MD[a], Konstantinos Theofilatos PhD[a], Anna Zampetaki PhD[a], Javier Barallobre-Barreiro PhD[a], Bhawana Singh MSc[a], Nils A Sörensen MD[b,c], Johannes T Neumann MD[b,c], Tanja Zeller PhD[b,c], Dirk Westermann MD[b,c], Stefan Blankenberg MD[b,c], Michael Marber MD[d], Christoph Liebetrau MD[e], Manuel Mayr MD, PhD[a]

a. King's British Heart Foundation Centre, King's College London, 125 Coldharbour Lane, London SE5 9NU, United Kingdom.
b. Department of General and Interventional Cardiology, University Heart Centre Hamburg Eppendorf, Hamburg, Germany
c. German Centre of Cardiovascular Research (DZHK), Partner Site Hamburg, Luebeck, Kiel, Hamburg, Germany
d. King's British Heart Foundation Centre, King's College London, Guy's & St Thomas' Hospitals, Lambeth Palace Rd, London SE1 7EH
e. Department of Cardiology, Kerckhoff Heart and Thorax Center, Bad Nauheim, Germany and German Centre of Cardiovascular Research (DZHK), Partner Site Rhine-Main, Bad Nauheim, Germany

Short title Circulating Biomarkers in Myocardial Injury

Corresponding Author Manuel Mayr, MD, PhD
King's British Heart Foundation Centre, King's College London
125 Coldharbour Lane, London SE5 NU
United Kingdom
Email: manuel.mayr@kcl.ac.uk

Subject Terms Biomarkers, Functional Genomics, Myocardial Infarction

Supplemental methods

Antibody-based measurements of cardiac proteins (cTnI, cTnT, cMyC)

All antibody-based measurements of cTnI, cTnT and cMyC were performed on assays proprietary to the manufacturers.

1) *Myocardial Tissue Spike-In*. The concentrations of cTnI and cTnT were measured using contemporary high-sensitivity assays [Abbott Architect, limit of detection (LoD) 1.9 ng/L; and Roche Elecsys, LoD 5 ng/L, respectively] as previously described[1]. cMyC was measured by EMD Millipore on the Erenna® platform using proprietary reagents as recently described (LoD of 0.4 ng/L)[1].

2) *TASH samples*. cMyC was quantified using an electrochemiluminescence (ECL) SECTOR imager 2400 instrument (MesoScale Discovery), by utilizing capture and detection monoclonal antibodies that recognized discrete epitopes on the C0C2 peptide as previously described [2]. In brief, the capture antibody (Clone 3H8/30 µL/1 µg/mL) was coated onto 96-well SECTOR® plates (MesoScale Discovery) in 10 mM Tris pH 9.6 overnight at 4°C, washed three times with PBS/0.05 % Tween, and blocked with 1 %BSA/PBS for 1 hour at room temperature on a platform shaker. Serum samples (30 µL/well) were diluted 1 in 2 with Diluent 7 (MesoScale Discovery) and were added to the plate along with recombinant C0C2 standards also diluted with Diluent 7, before incubation for 1 h at room temperature. Afterwards, the plates were washed three times with PBS/0.05 % Tween to reduce non-specific binding. Detection antibody (Clone 1A4/30 µL/PBS pH 7.4), which had been conjugated to ruthenium (MesoScale Discovery) according to the manufacturer's instructions, was then added to the wells and incubated for 2 h at room temperature with shaking at 300 rpm. Finally, the plates were washed three times with PBS/0.05 % Tween, and 150 µL 1× read buffer was added to the wells prior to ECL analysis on the SECTOR imager 2400. The standard curve generated was then used to quantify the cMyC concentration present in serum samples and expressed as ng/L. hs-cTnT measurements were performed as previously described[3]. In brief, hs-cTnT was measured in serum with the hs-electrochemiluminescence immunoassay (hs-cTnT assay, Elecsys Analyzer 2010, Roche Diagnostics). The lower detection limit for the hs-cTnT assay is 3.0 ng/L, with the 99th percentile at a concentration of 14.0 ng/L.

3) *MI cohort (BACC)*. cMyC was measured by Merck Millipore using the Erenna platform with a lower limit of detection (LLoD)of 0.4 ng/L and a lower limit of quantification (LLoQ of 1.2 ng/L. The 99th percentile cut-off point was previously determined at 87 ng/L[4]. Troponin was routinely measured using the local standard of care troponin T assay (Elecsys® troponin T high-sensitive, Roche Diagnostics, Basel, Switzerland, lower limit of detection 5.0ng/L, values are reported up until the limit of blank 3.0ng/L) at admission and after 3 hrs. A study specific additional blood draw was ascertained 1 hour after admission and for troponin I (Troponin I hs STAT Abbott Arcitect, lower limit of detection 1.9ng/L) at all time points. The 99th percentile is set to 14ng/L (hs-cTnT) and 27ng/L (hs-cTnI) respectively[5,6].

Measurements of cTnI by proximity extension assays (PEA)

In order to quantify cardiac troponin I (cTnI) in TASH plasma samples using a qPCR-based method, proximity extension assays (PEA) were performed as previously published[7,8]. cTnI was part of the 'organ damage panel' from Olink (Uppsala, Sweden).

RNA extraction quality control

In order to assess the reliability and efficiency of the RNA extractions Cel-miR-39 spike-in raw Cq values were plotted for TASH and MI cohort (**Supplemental Figure 18**). The standard deviation is below 0.5 and the coefficient of variation below 2% in both groups, confirming good RNA processing and consistency of the RNA isolation.

Reverse transcription

Analysis of miRNAs. 3 µl of RNA from plasma/serum RNA was used as input in each reverse transcription (RT) reaction. RT reactions were set up according to the manufacturer's recommendations. Briefly, miRNAs were reverse-transcribed using the miRCURY LNA RT kit (Exiqon), combining 3µl RNA with 5x reaction buffer, 1µl enzyme mix, 0.5µl UniSp6 synthetic spike-

in and 3.5µl nuclease-free water. The RT-PCR reaction was set as follows: reverse transcription, 42°C for 60 minutes; inactivation, 95°C for 5 minutes using a Veriti Thermal Cycler (Applied Biosystems).

Analysis of lncRNAs and circRNAs. RT was performed using the SuperScript VILO cDNA Synthesis Kit (Invitrogen). 2 µl of VILO RT Master Mix were combined with 8 µl of sample. Thermal cycler stages were set as follows: incubation at 25°C for 10 minutes and synthesis at 42°C for 120 minutes, followed by termination of the reaction at 85°C for 5 minutes. cDNA products were stored at -20ºC.

Real-time PCR assays

Custom-designed primers for detection of lncRNAs and circRNAs were produced by IDT Oligos (Integrated DNA Technologies, Inc., 8180 N. McCormick Blvd., Skokie, Illinois 60076, USA). A list of primers used for qPCR detection and their sequence is provided in **Supplemental Table 1**. miRCURY SYBR Green qPCR in combination with miRCURY LNA miRNA PCR Assays (for miRNAs, both Exiqon) and SYBR Select Master Mix (Applied Biosystems) in combination with custom-made primers were used to assess relative expression levels of miRNAs and lncRNAs/circRNAs, respectively. For miRCURY SYBR Green, cDNA was diluted 1:30 according to the manufacturer's recommendations, then 3µl of the diluted cDNA were combined with 5µl miRCURY SYBR Green Mastermix, 0.05µl ROX reference dye, 1µl PCR Primer Mix and 0.95µl of RNase-free water to a 10µl reaction volume. For SYBR Select Master Mix, cDNA was diluted 1:20, then 2µl of diluted cDNA were combined with 2.5µl of SYBR Select Master Mix and 0.1µl of 10µM forward primer and 0.1µl of 10µM reverse primer. Reactions were loaded using a Bravo Automated Liquid Handling Platform (Agilent). qPCR was performed on a ViiA7 Real-Time PCR System (Applied Biosystems) at 95ºC for 2 minutes followed 40 cycles of 95ºC for 10 seconds and 56ºC for 1 minute for miRCURY SYBR Green (miRNA) and at 50 ºC for 2 minutes, then 95ºC for 2 minutes, followed by 40 cycles of 95ºC for 15 seconds and 60ºC for 1 minute for SYBR Select Master Mix (lncRNAs and circRNAs), respectively.

miRNA detectability Cq cut-off determination

In order identify unreliable measurements of miRNAs we set a Cq threshold of 35. The threshold was set so that we exclude Cq values which follow uniform distribution and can thus be considered random noise. To make these calculations we used the one-way Kolmogorov-Smirnov test for all cohorts of this paper. For all four muscle/cardiac-enriched miRNAs of interest the p-value of the test for the Cq values greater than 35 was smaller than 0.05 and bigger than 0.05 for the Cq values smaller than 35.

Selection of appropriate miRNA normalisation method

In order to identify potential endogenous candidates to be used as normalisation controls, we analysed expression levels of some of the most abundant plasma miRNAs in the TASH cohort. None of them returned expression profiles, which can be considered stable enough for use as normalisation controls, especially compared with Cel-miR-39 which was extremely stable justifying our decision for using it (**Supplemental Figure 18 and 19**). To further assess the validity of this proposal we re-conducted normalization using the average of all non-coding RNAs instead of Cel-miR-39 as normalizer. For the four central miRNAs of our interest we conducted correlation analysis between the RQ values obtained from its one of the normalization strategies and in all cases we found strong correlation (R2>0.5) indicating that the selection of the normalization method has no relevant influence on the overall RQ results.

Myocardial tissue spike-in experiment

In order to assess the detectability of RNAs with cardiac origin, different amounts of human cardiac tissue were spiked into human plasma before extracting RNA. Tissue was lysed in Qiazol at a concentration of 1µg/µl. Lysis was performed in a FastPrep-24 Homogeniser (MP Biomedicals) at 6000 rpm for two rounds of 20 seconds using Lysing Matrix D beads (MP Biomedicals). Then the desired concentration was added to the RNA extraction process as described above using 100µl of plasma from healthy volunteers (**Supplemental Figure 1**). For protein analyses, human myocardium was weighed, and the tissue was crushed in a percussion mortar for 10 seconds as previously described[1]. Buffer

solution [50 mL Tris, pH 7.5, containing a protease inhibitor cocktail (cOmplete EDTA-free, Roche)] was added to the pulverized tissue (1 mL of buffer per 100 mg of tissue). The subsequent solution was subject to ultrasonication on ice (6 × 10-second bursts on ice, with 10-second intervals on ice). Following ultrasonication, the solution was centrifuged at 21,130g for 30 minutes at 4 °C. The supernatant was frozen in liquid nitrogen and then stored at −80 °C. Dilutions of this solution were then spiked into 400µL of human serum.

Microarray-based screening of circRNAs in TASH

To widen our search for cardiac-enriched circRNAs that are detectable in plasma, we performed a microarray-based screening of circRNAs in pooled TASH plasma samples (n=4 per time point) screening for 13,617 circRNAs: Total RNA from each TASH sample was quantified using spectrophotometry (NanoDrop ND-1000). The integrity of RNA was assessed by electrophoresis on a denaturing agarose gel. The sample preparation and microarray hybridization were performed based on the Arraystar's (Arraystar, Inc., Rockville, MD, USA) standard protocols as previously described (Huang et al., 2017). Briefly, total RNAs were digested with Rnase R (Epicentre, Inc.) to remove linear RNAs and enrich circular RNAs. Then, the enriched circRNAs were amplified and transcribed into fluorescent cRNA utilizing a random priming method (Arraystar Super RNA Labeling Kit; Arraystar). The labeled cRNAs were hybridized onto the Arraystar Human circRNA Array v2 (8x15K, Arraystar). After having washed the slides, the arrays were scanned by the Agilent Scanner G2505C. Agilent Feature Extraction software (version 11.0.1.1) was used to analyze acquired array images. Quantile normalization and subsequent data processing were performed using the R software limma package. Differentially expressed circRNAs with statistical significance between two groups were identified through Volcano Plot filtering. Differentially expressed circRNAs between two samples were identified through Fold Change filtering. Hierarchical Clustering was performed to show the distinguishable circRNAs expression pattern among samples. Detectability was defined as present or marginal in at least three or more of 16 TASH plasma samples. 6812 circRNAs were detectable in plasma of TASH patients. Of these, 4106 circRNAs showed an increase in expression levels between time point 0 hours and 1 hour, whilst 4225 and 4255 circRNAs showed an increase between 0 hours and 8 hours and between 0 hours and 24 hours, respectively. Anova test was used to determine statistically significant changes in circRNAs in the TASH cohort. Correction for multiple testing was conducted with Benjamini-Hochberg method. None of the circRNAs was significantly regulated with an FDR threshold of 10% **(Supplemental Figure 9).**

Myocardial tissue

For cardiac tissue spike-in analyses and primer testing, samples from human myocardial tissue were obtained from an explanted failing heart under Ethical Approval from the Royal Brompton and Harefield Trust BRU Biobank and from nonfailing hearts under Ethical Approval approval from the local Research Ethics Committee and institutional Research and Development office for St George's Hospital and King's College London. The study was performed in accordance with the Declaration of Helsinki. All patients gave written informed consent. All samples were frozen at −80 °C.

TASH cohort

The TASH cohort patient characteristics and procedure as well as the blood sampling methods have previously been described[3]. Samples from 20 patients undergoing this procedure were chosen on the basis of adequate sample volumes and complete data sets from four different time points (baseline, 1 h, 8 hrs and 24 hrs after onset of myocardial injury). For ncRNA analyses samples from a subset of 16 patients were available. Hs-cTnT and cMyC data were available from 15 and 20 patients, respectively. For clinical assessment in TASH, those circRNAs and lncRNAs with Cq<25 cycles in cardiac tissue were selected for further analyses, that were detectable in >50% of 12 pooled TASH samples or detectable in all samples of one time point (n=3 per time point) (**Supplemental Figure 20).** Results from the evaluation of the selected circRNAs and lncRNAs are depicted in **Supplemental Figure 21 and 22**, respectively. Only, circSMARCA and circPCMTDL were detectable in >50% per time point, and therefore evaluated in terms of kinetics.

The Biomarkers in Acute Cardiac Care (BACC) study

The BACC study has been described before[9]. Briefly, the study prospectively included 2,335 patients presenting to the emergency department and chest pain unit of the University Hospital Hamburg. The inclusion criteria were suspected acute MI, age above 18 and the ability to provide written informed consent. All patients were triaged according to local standard of care: A standard ECG was collected at admission. Troponin was routinely measured using the local standard of care troponin T assay (Elecsys® troponin T high-sensitive, Roche Diagnostics, Basel, Switzerland, LLoD 5.0ng/L, values are reported up until the limit of blank 3.0ng/L) at admission and after 3 hrs. A study-specific additional blood draw was ascertained 1 hour after admission and for troponin I (Troponin I hs STAT Abbott Arcitect, LLoD 1.9ng/L) at all time points. The 99th percentile is set to 14ng/L (hs-cTnT) and 27ng/L (hs-cTnI) respectively[5,6].

The self-reported onset of pain was obtained from a study-specific questionnaire or medical records and then categorised to time intervals as follows: 0-1 h, 1-3 hrs, 3-6 hrs, 6-12 hrs, 12-24 hrs, 24-72 hrs. The final diagnosis was adjudicated by two cardiologists independently, taking to account the troponin T results (Roche) and all available clinical and imaging results, ECG and routine laboratory testing. In cases of disagreement, a third cardiologist reviewed the case. A subset of patients was selected for this study, excluding NSTEMI Type 2 patients and balancing patients with STEMI (n=20) and NSTEMI (n=18). The BACC study was registered at www.clinicaltrials.gov (NCT02355457), complied with the Declaration of Helsinki and was approved by the local Ethics Committee.

Predictive analytics with TASH and BACC cohorts

Quantitative values of both miRNAs and proteins from samples of TASH and BACC cohorts were arithmetically normalized to range in the interval [0,1]. Missing values were imputed using the K-Nearest Neighbours imputation method[10] with k=20 (default value).

The markers measured in TASH cohort, were combined considering all meaningful combinations and using the Support Vector Regression method[11] to construct regression models which can predict a score that corresponds to the time passed in hours from the onset of the injury. Non-linear Radial Basis Functions and linear kernels[12] were explored as the more suitable kernel functions to be used for Support Vector Machines.

The Nondominated Sorting Genetic Algorithm (NSGA) optimization method[13] was used to select the optimal kernel function and to tune the Regularization parameter C of SVM and the gamma parameter of Radial Basis Functions. The parameters used for the NSGA algorithm were: Population Size: 50, Maximum Number of Generations: 200, Crossover probability: 90%, Mutation probability: 1%. Three different competitive optimization goals were set to guide the optimization function, two related with the prediction accuracy and one related with the complexity of the prediction model. These goals were formulated to the following fitness functions:

- Fitness function 1: 1/(1+Root Mean Square Error)
- Fitness function 2: Squared correlation coefficient
- Fitness function 3: Number of Samples of the Dataset/Number of Support Vectors of Trained Model

For the evaluation of the individual models we utilized the leave-one-out cross validation approach[14]. The libSVM implementation[15] was used for training and testing the SVR models and for the NSGA algorithm an open python implementation was used (https://github.com/haris989/NSGA-II)

TASH cohort was used as the training cohort and the fitness functions were measured using the leave one-out-approach. BACC cohort was used as a validation cohort. The most promising predictive models according to their performances in the TASH dataset were applied on the BACC dataset without retraining them for this dataset to measure the generalization performance of the trained model on an independent validation cohort. ROC curves analysis was conducted using the LABROC4 algorithm[16].

Online References

1. Marjot J, Kaier TE, Martin ED, Reji SS, Copeland O, Iqbal M, Goodson B, Hamren S, Harding SE, Marber MS. Quantifying the Release of Biomarkers of Myocardial Necrosis from Cardiac Myocytes and Intact Myocardium. *Clin. Chem.* 2017;63(5):990–996.
2. Baker JO, Tyther R, Liebetrau C, et al. Cardiac myosin-binding protein C: a potential early biomarker of myocardial injury. *Basic Res. Cardiol.* 2015;110(3):23.
3. Liebetrau C, Möllmann H, Nef H, Szardien S, Rixe J, Troidl C, Willmer M, Hoffmann J, Weber M, Rolf A, Hamm C. Release kinetics of cardiac biomarkers in patients undergoing transcoronary ablation of septal hypertrophy. *Clin. Chem.* 2012;58(6):1049–54.
4. Marjot J, Liebetrau C, Goodson RJ, Kaier T, Weber E, Heseltine P, Marber MS. The development and application of a high-sensitivity immunoassay for cardiac myosin-binding protein C. *Transl. Res.* 2016;170:17–25.e5.
5. Zeller T, Ojeda F, Brunner FJ, Peitsmeyer P, Münzel T, Binder H, Pfeiffer N, Michal M, Wild PS, Blankenberg S, Lackner KJ. High-sensitivity cardiac troponin I in the general population – defining reference populations for the determination of the 99th percentile in the Gutenberg Health Study. *Clin. Chem. Lab. Med.* 2015;53(5):699–706.
6. Westermann D, Neumann JT, Sörensen NA, Blankenberg S. High-sensitivity assays for troponin in patients with cardiac disease. *Nat. Rev. Cardiol.* 2017;14(8):472–483.
7. Assarsson E, Lundberg M, Holmquist G, et al. Homogenous 96-plex PEA immunoassay exhibiting high sensitivity, specificity, and excellent scalability. *PLoS One*. 2014;9(4):e95192.
8. Kaudewitz D, Skroblin P, Bender LHLH, et al. Association of MicroRNAs and YRNAs With Platelet Function. *Circ. Res.* 2016;118(3):420–32.
9. Neumann JT, Sörensen NA, Schwemer T, et al. Diagnosis of Myocardial Infarction Using a High-Sensitivity Troponin I 1-Hour Algorithm. *JAMA Cardiol.* 2016;1(4):397.
10. Troyanskaya O, Cantor M, Sherlock G, Brown P, Hastie T, Tibshirani R, Botstein D, Altman RB. Missing value estimation methods for DNA microarrays. *Bioinformatics*. 2001;17(6):520–5.
11. Smola, A. J., & Schölkopf, B. (2004). A tutorial on support vector regression. Statistics and computing, 14(3), 199-222.
12. Vert, J. P., Tsuda, K., & Schölkopf, B. (2004). A primer on kernel methods. Kernel methods in computational biology, 47, 35-70.
13. Deb, K., Pratap, A., Agarwal, S., & Meyarivan, T. A. M. T. (2002). A fast and elitist multiobjective genetic algorithm: NSGA-II. IEEE transactions on evolutionary computation, 6(2), 182-197.
14. Wong, T. T. (2015). Performance evaluation of classification algorithms by k-fold and leave-one-out cross validation. Pattern Recognition, 48(9), 2839-2846.
15. Chang, C. C., & Lin, C. J. (2011). LIBSVM: a library for support vector machines. ACM transactions on intelligent systems and technology (TIST), 2(3), 27.
16. Metz CE, Herman BA, Shen JH. Maximum likelihood estimation of receiver operating characteristic (ROC) curves from continuously-distributed data. *Stat. Med.* 1998;17(9):1033–53.

Supplemental Figures

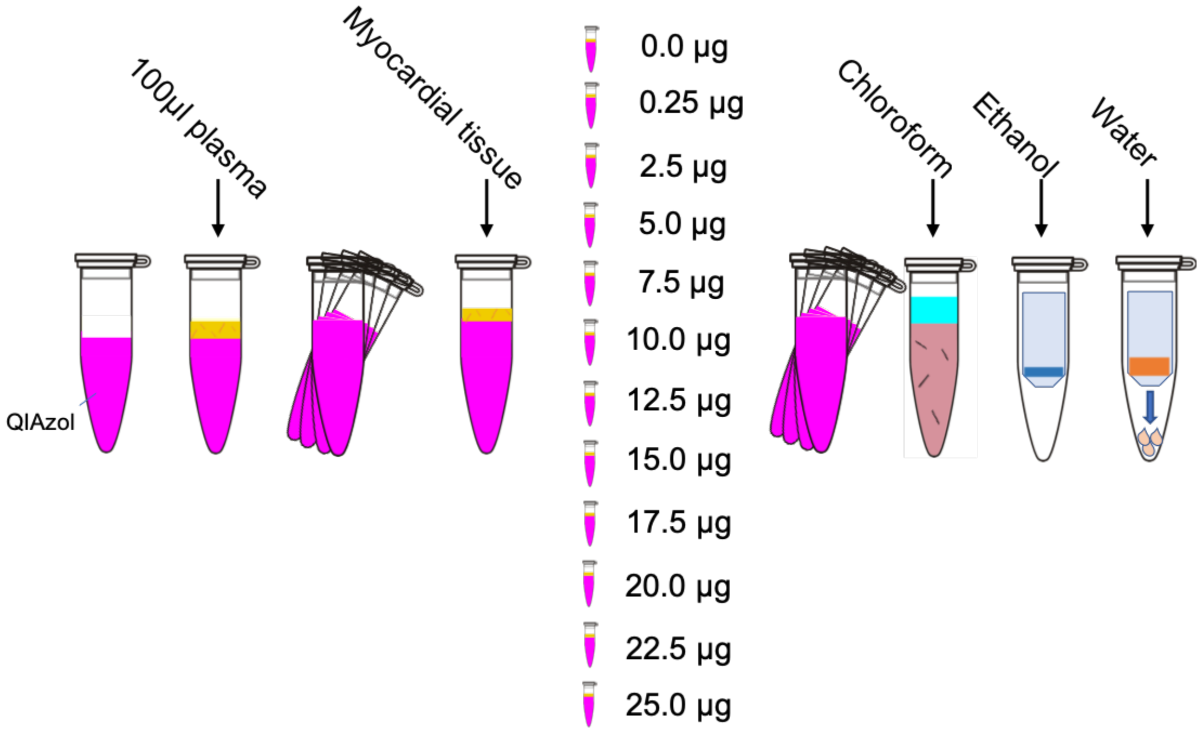

Online Figure I. Workflow of myocardial tissue spike-in. 100µl of plasma from healthy control individuals was first spiked into Qiazol lysis buffer (Qiagen, Hilden, Germany) and shaken vigorously before human cardiac tissue (also already lysed in Qiazol) was added at different concentrations and again shaken vigorously. RNA extraction was then performed according to the manufacturer's recommendations and as described in the methods section.

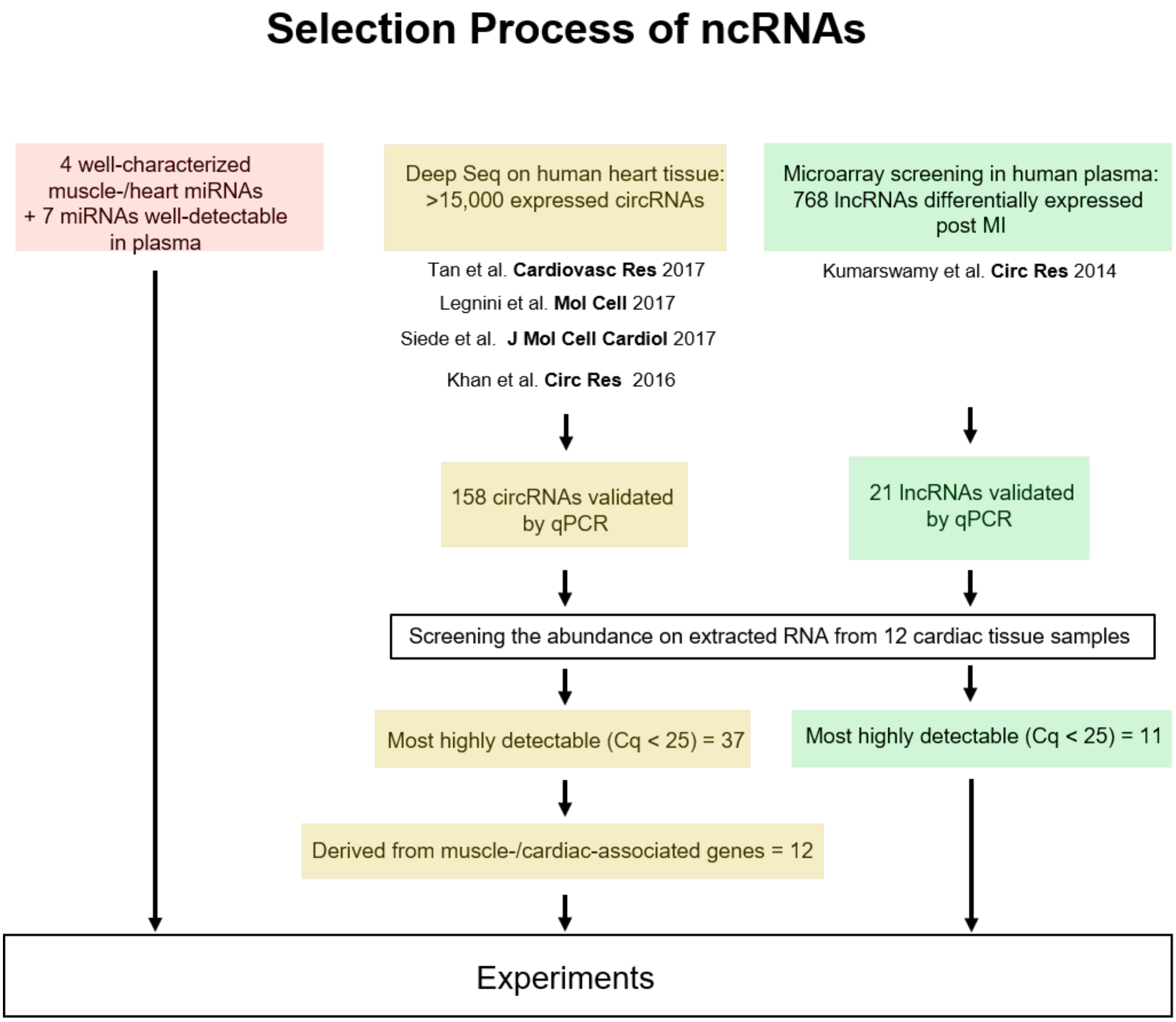

Online Figure II. Selection process of ncRNAs used in the experiments. *miRNAs* have previously been studied in the context of myocardial injury, and we selected muscle-/cardiac miRNAs plus some other miRNAs that are well detectable in human plasma. *circRNAs* are the least well-studied class of ncRNAs. We interrogated the literature for deep sequencing data of human cardiac tissue and selected the circRNAs that were reported to be detectable via qPCR. *circRNA* primers were further selected for the muscle-/cardiac specificity of their related gene. *lncRNAs* were chosen from published microarray data on human cardiac tissue and selected based on qPCR validation. Both, custom-made *circRNA* and *lncRNA* primers were tested on extracted RNA from human cardiac tissue; only the most abundant RNAs in myocardial tissue were chosen for further analyses in human plasma and serum.

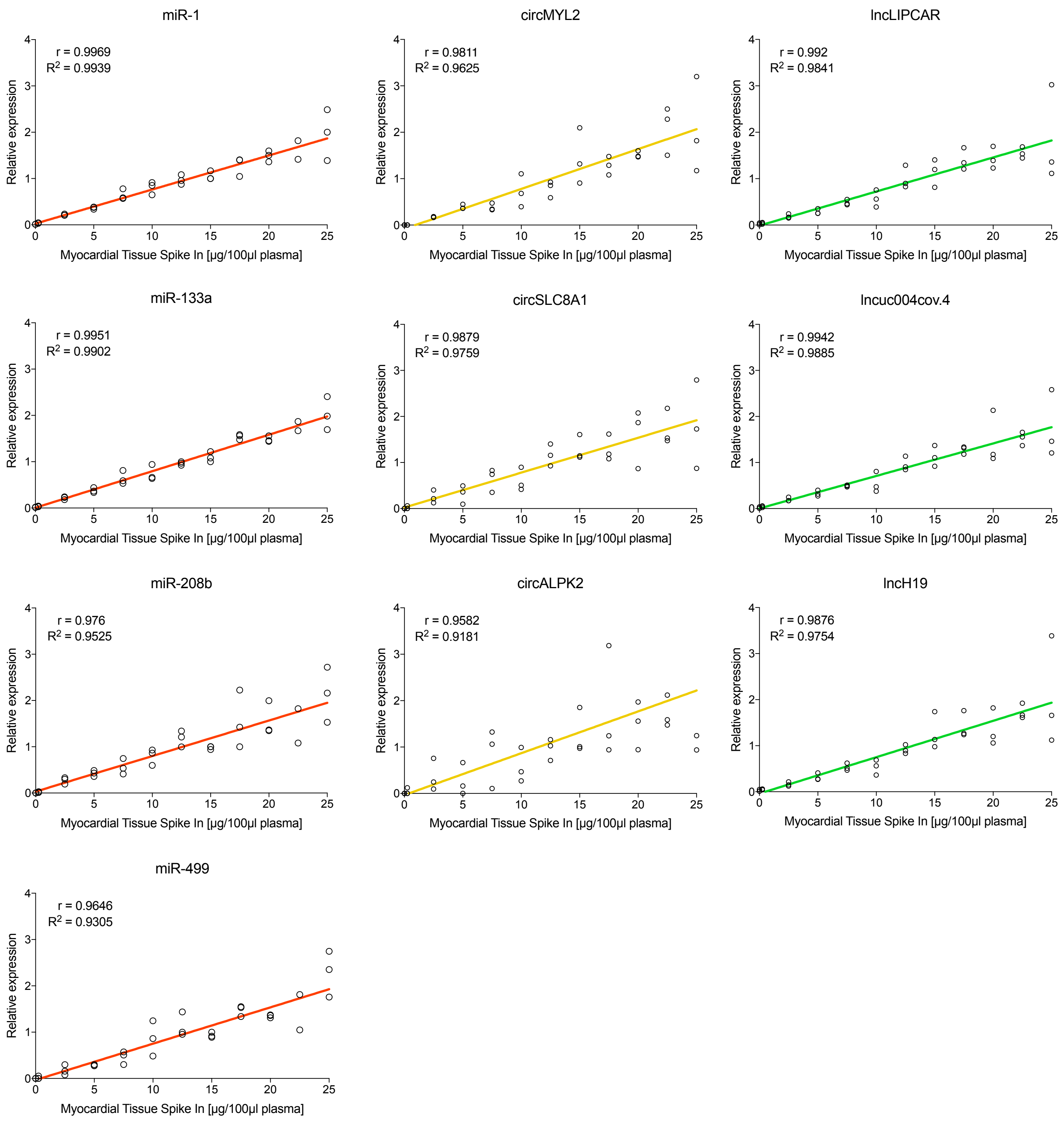

Online Figure III. Myocardial tissue spike-In. Regression curves for a selection of ncRNA biomarkers with the highest r and R^2 values. Left Y-axis depicts relative expression referenced to the individual biomarker's median relative expression value. r = Pearson correlation coefficient; R^2 = Coefficient of determination.

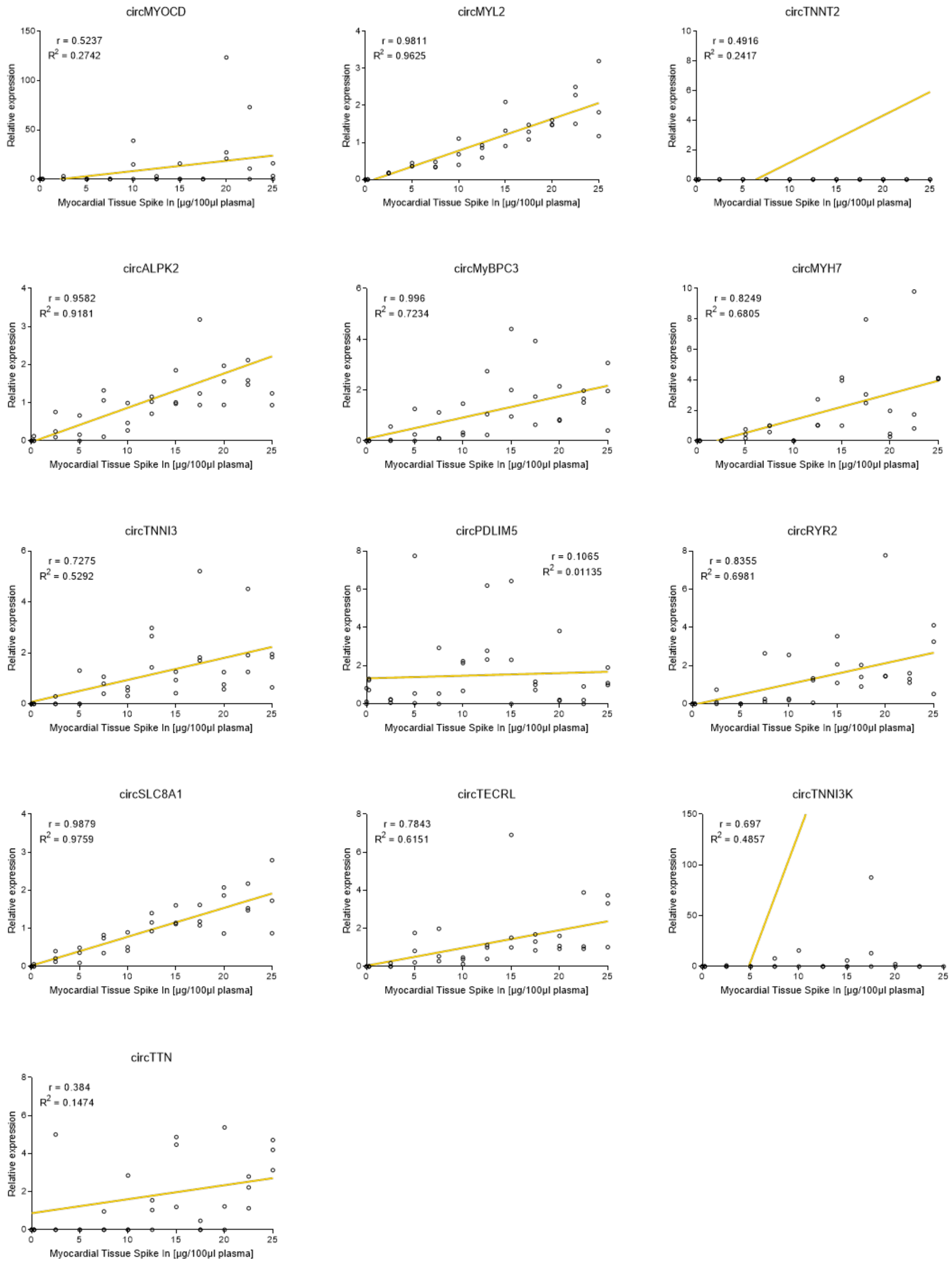

Online Figure IV. circRNAs in myocardial tissue spike-in. Regression curves for all circRNA biomarkers tested in myocardial spike-in. Left Y-axis depicts relative expression referenced to the individual biomarker's median relative expression value. r = Pearson correlation coefficient; R^2 = Coefficient of determination

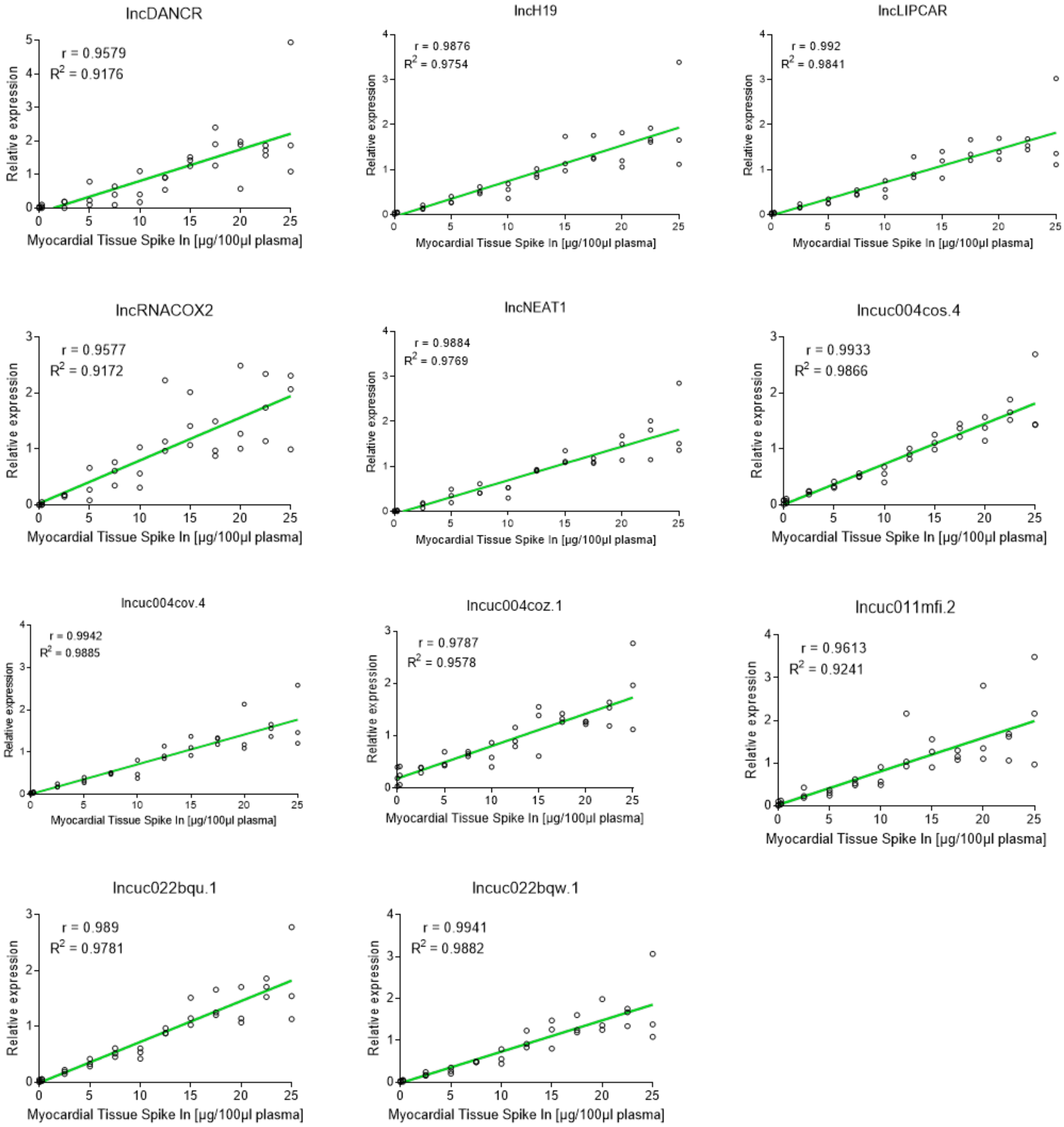

Online Figure V. lncRNAs in myocardial tissue spike-in. Regression curves for all lncRNA biomarkers tested in myocardial spike-in. Left Y-axis depicts relative expression referenced to the individual biomarker's median relative expression value. r = Pearson correlation coefficient; R^2 = Coefficient of determination

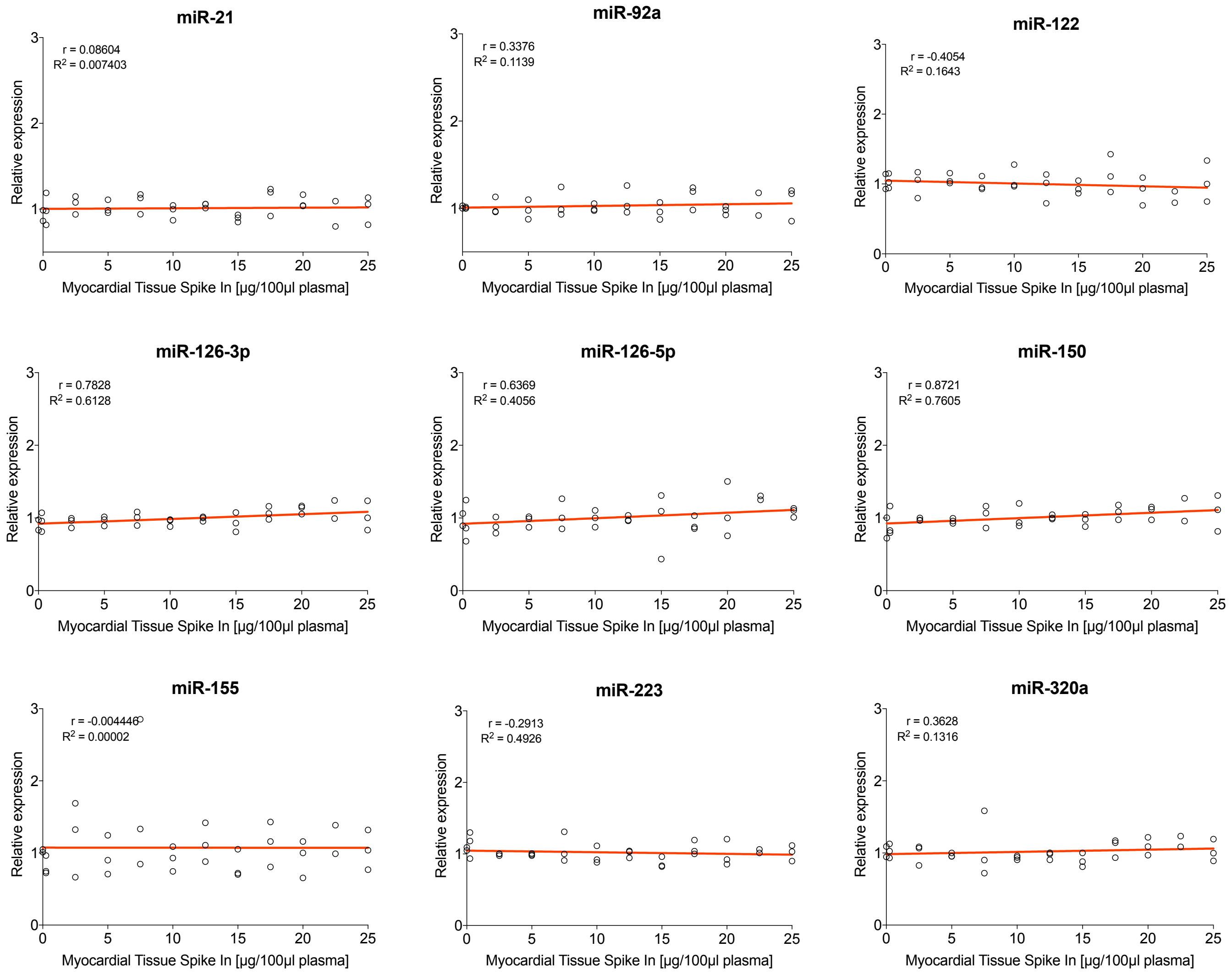

Online Figure VI. Non muscle-/cardiac enriched miRNAs in myocardial tissue spike-in. All miRNAs not enriched in muscle or myocardium fail to show a relevant increase in human plasma after myocardial tissue spike-in in different concentrations. r = Pearson's correlation coefficient, R^2 = Coefficient of determination.

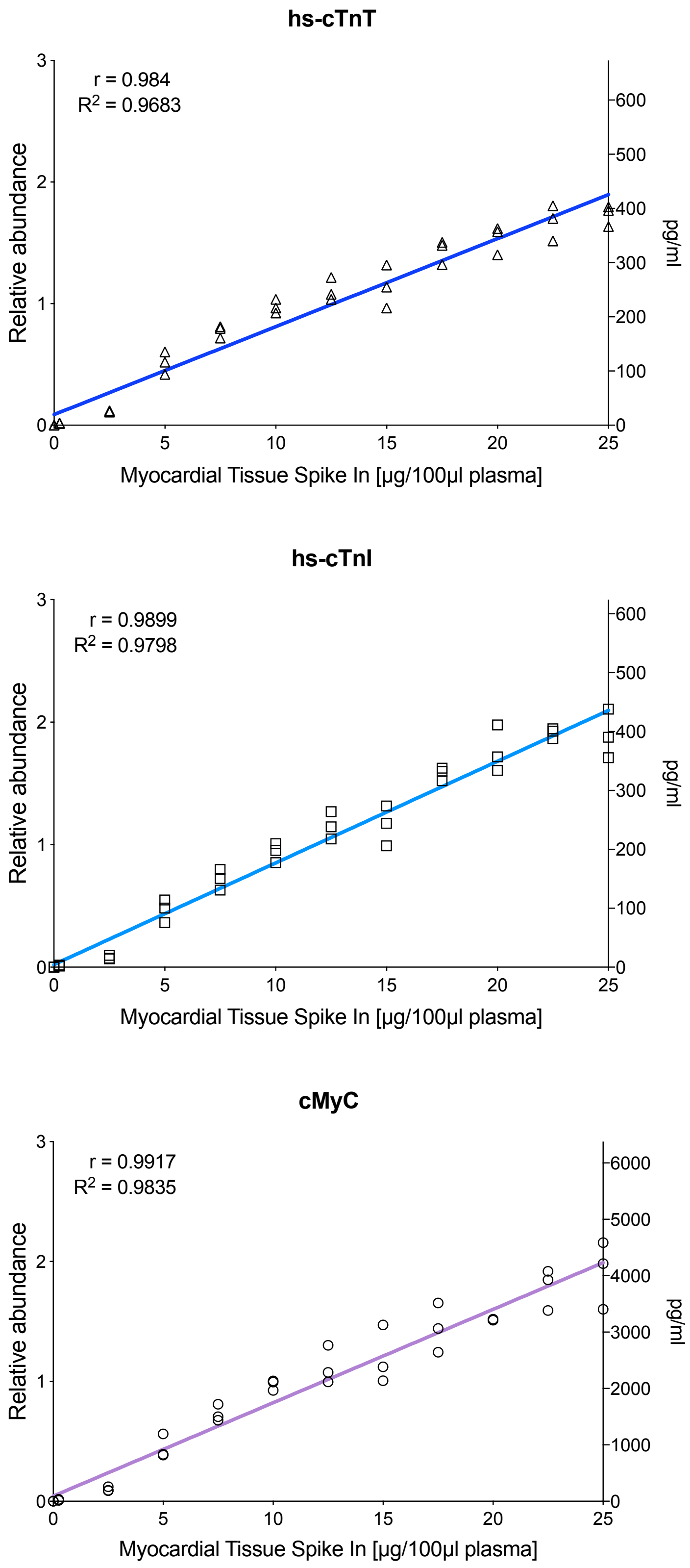

Online Figure VII. Myocardial tissue spike-in for protein biomarkers. Regression curves for cardiac protein biomarkers. Left Y-axis depicts relative expression referenced to the individual biomarker's median value. Right Y-axis depicts the absolute concentration in pg/ml. cMyC = cardiac myosin binding protein C, hs-cTnT = high sensitive cardiac troponin T; hs-cTnI = high sensitive cardiac troponin I; r = Pearson correlation coefficient; R^2 = Coefficient of determination.

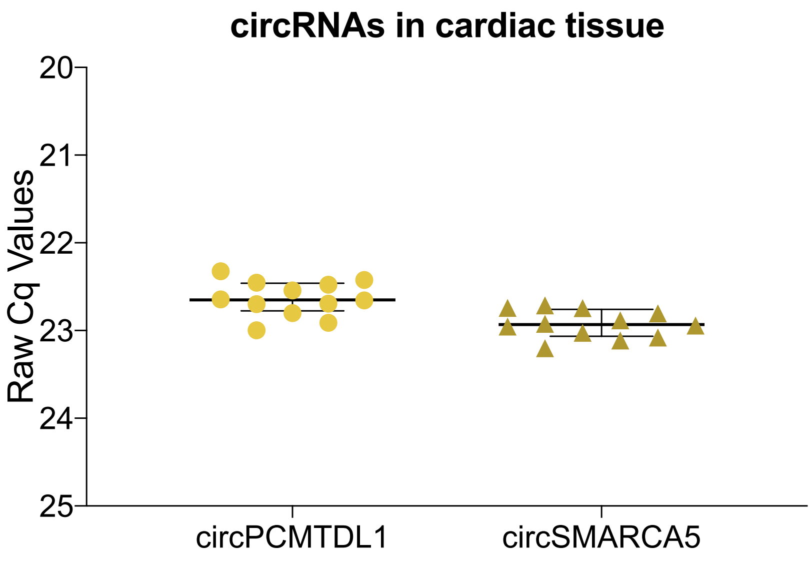

Online Figure VIII. Raw Cq-values of selected circRNAs in human cardiac tissue. circPCMTDL1 and circSMARCA5 were detectable in 12 human cardiac tissue samples. Their expression was stable and abundant based on raw Cq values.

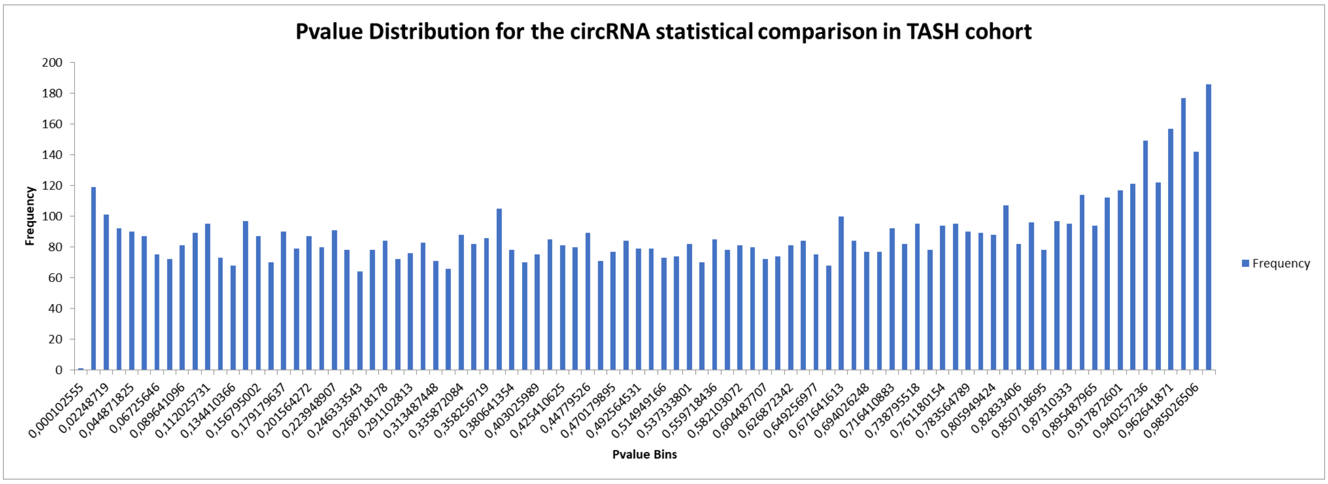

Online Figure IX. **P-value distribution for the statistical comparison of changes in circRNAs after TASH.** An Anova test was used to test for statistically significant changes of circRNAs at 0, 4, 8 and 24 hours after TASH (n=4 pools at each time point, respectively). Correction for multiple testing was performed with Benjamini-Hochberg adjustment. None of the circRNAs was found significant with an FDR threshold of 10%. The distribution of the p-values after correcting for multiple testing, which would we expected to be skewed towards the 0 value of the x-axis in case of true significance. This is not the case, indicating that all initial significant changes are lost.

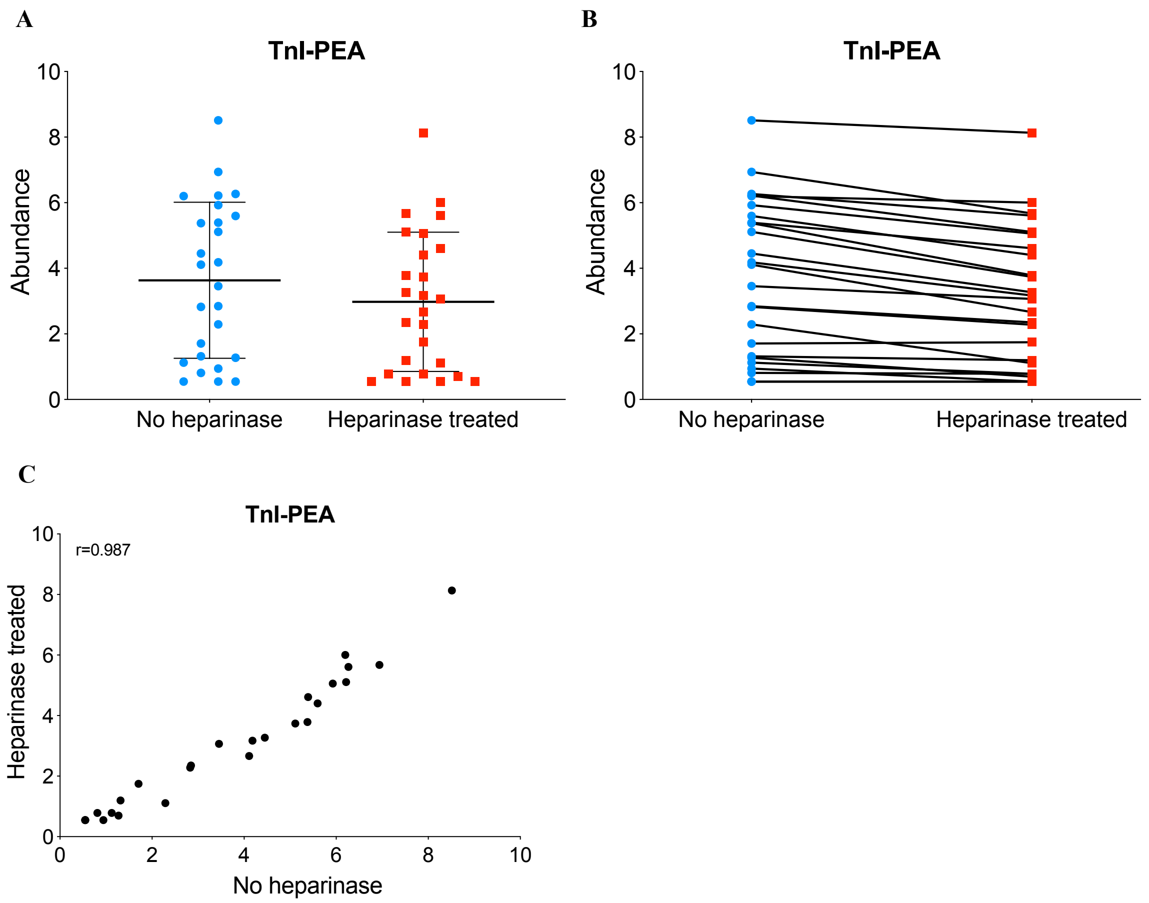

Online Figure X. Comparison of PEA data in heparin and non-heparin treated samples from the TASH cohort. As expected, after heparinase treatment slightly lower overall values were measured, caused by the dilution effect (**A** and **B**). Values between the two groups were highly correlated (**C**). PEA = Proximity Extension Assay (Olink).

A

	hs-cTnT	hs-cTnI	cMyC	TnI-PEA	CK-MB	CK	miR-1	miR-133a	miR-208b	miR-499
**hs-cTnT**	1,0000	0,9559	0,9237	0,9022	0,9255	0,8767	0,7485	0,8426	0,8661	0,9282
**hs-cTnI**		1,0000	0,9015	0,9012	0,9216	0,8448	0,6719	0,7900	0,8193	0,8457
**cMyC**			1,0000	0,9266	0,8513	0,8735	0,8079	0,8699	0,8530	0,8776
**TnI-PEA**				1,0000	0,8239	0,8630	0,7344	0,8069	0,7721	0,7672
**CK-MB**					1,0000	0,9585	0,7571	0,8562	0,8610	0,8890
**CK**						1,0000	0,7912	0,8076	0,8767	0,8886
**miR-1**							1,0000	0,9028	0,8796	0,8649
**miR-133a**								1,0000	0,9086	0,9347
**miR-208b**									1,0000	0,9472
**miR-499**										1,0000

B

	hs-cTnT	hs-cTnI	cMyC	TnI-PEA	CK-MB	CK	miR-1	miR-133a	miR-208b	miR-499
**hs-cTnT**	1,0000	0,9583	0,9282	0,9133	0,8785	0,7668	0,7177	0,7460	0,8106	0,8773
**hs-cTnI**		1,0000	0,9287	0,9230	0,8925	0,7689	0,6789	0,7107	0,7503	0,7941
**cMyC**			1,0000	0,9262	0,8681	0,7804	0,7924	0,7899	0,8002	0,8748
**TnI-PEA**				1,0000	0,8749	0,8128	0,7609	0,7588	0,7888	0,8046
**CK-MB**					1,0000	0,8922	0,7321	0,8355	0,8148	0,8249
**CK**						1,0000	0,6826	0,6733	0,7943	0,8202
**miR-1**							1,0000	0,8653	0,7843	0,8350
**miR-133a**								1,0000	0,8391	0,8787
**miR-208b**									1,0000	0,9118
**miR-499**										1,0000

Online Figure XI. Correlation of cardiac biomarkers in patients with STEMI and NSTEMI Type 1. All analysed biomarkers are highly correlated. Cardiac-enriched miRNAs correlated better with hs-cTnT and among each other than with muscle-enriched miRNAs. These correlations were higher in patients with STEMI (**A**) than in patients with NSTEMI Type 1 (**B**). Depicted are regression coefficients; p for all combinations <0.0001. CK = Creatine kinase; CK-MB = Creatine kinase muscle/brain; cMyC = cardiac myosin-binding protein C; Hs-cTnI = high sensitive cardiac troponin I; hs-cTnT = high sensitive cardiac troponin T, NSTEMI = non-ST elevation myocardial infarction; STEMI = ST elevation myocardial infarction; TnI-PEA = cardiac troponin I as measured by a proximity extension assay (PEA, Olink).

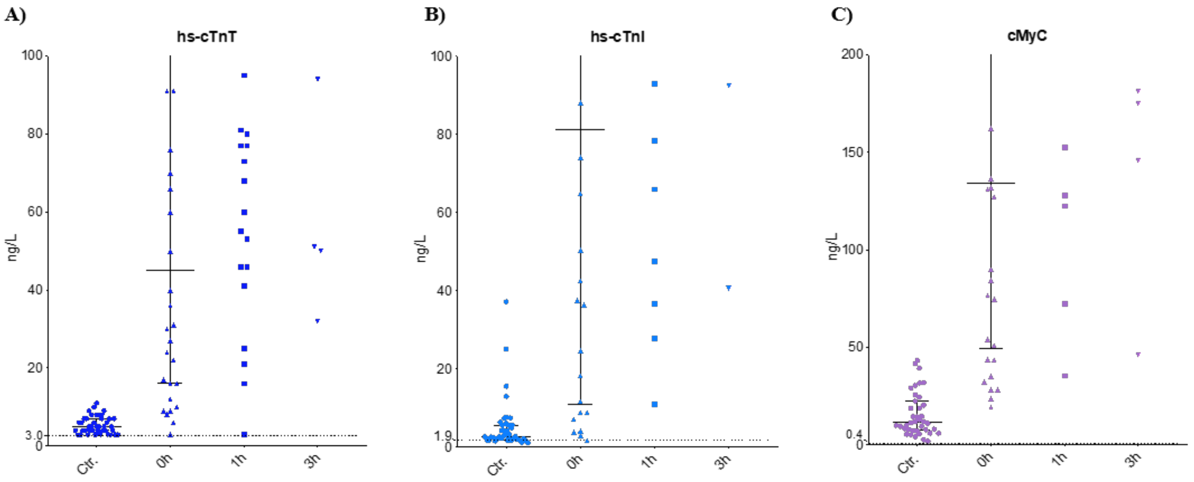

Online Figure XII. Release kinetics of cardiac protein biomarkers in the acute MI cohort. Values are depicted only for levels up to 100ng/L (troponins) and 200ng/L (cMyC) in order to facilitate visualization of the lower concentration range. Cardiac troponins and cMyC are depicted in control patients (Ctr.) vs. acute MI patients at hospital presentation (0h) and 1 hour and 3 hours after. Diagnosis of MI is adjudicated based upon hs-cTnT. hs-cTnT (**A**) detects all patients with acute MI at 3h after admission. Data for hs-cTnI (**B**) and cMyC (**C**) are shown in comparison. The black dotted line indicates the lower limit of detection (LLoD). hs-cTnT = high-sensitive cardiac troponin T; Hs-cTnI = high-sensitive cardiac troponin I; cMyC = cardiac myosin-binding protein C.

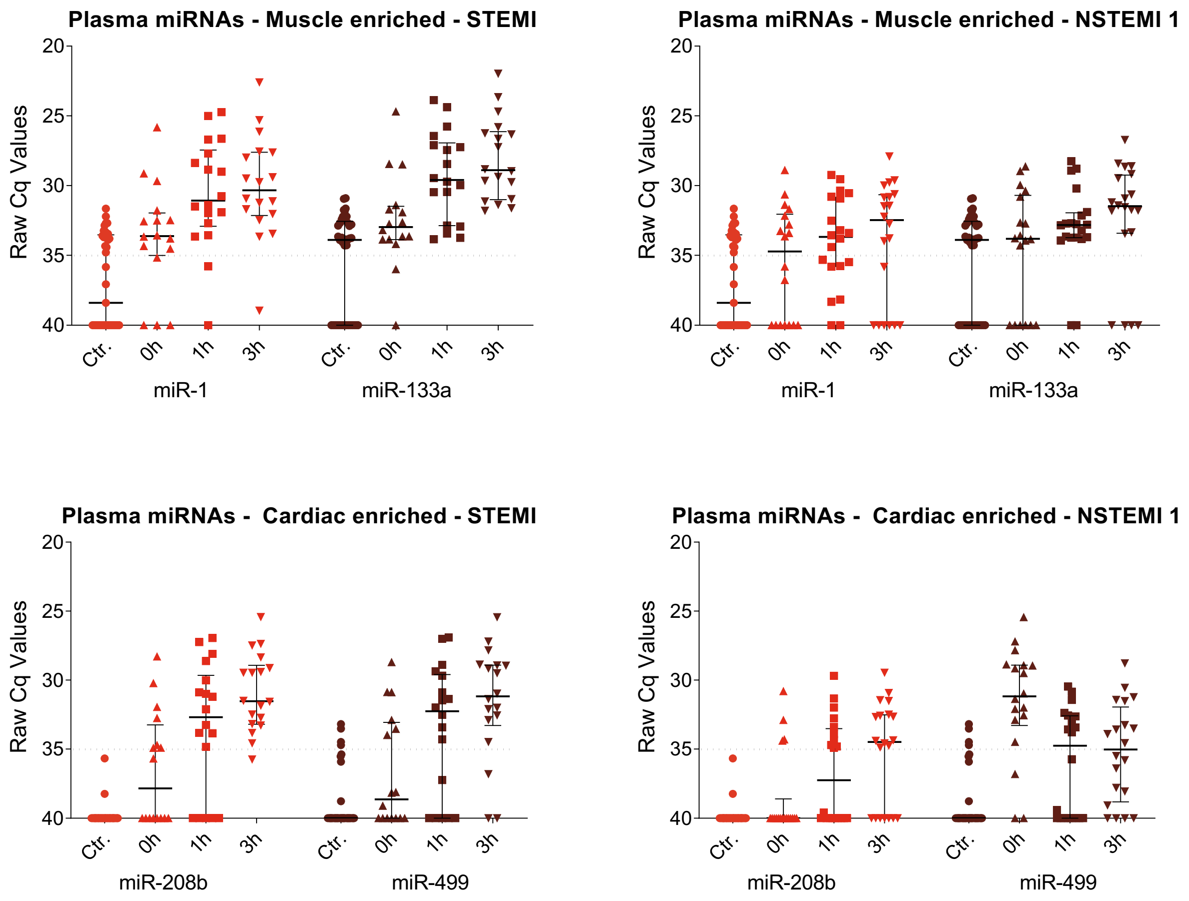

Online Figure XIII. miRNA raw expression data in the MI cohort. Raw Cq values of miRNAs stratified for STEMI and NSTEMI Type 1 patients compared with control patients (Ctr.) according to time of admission to hospital: 0h on presentation at hospital, 1 h and 3 hrs after presentation. At every time point, there were undetectable values (Cq>35) for each miRNA. This is more pronounced in NSTEMI Type 1 patients compared with STEMI patients.

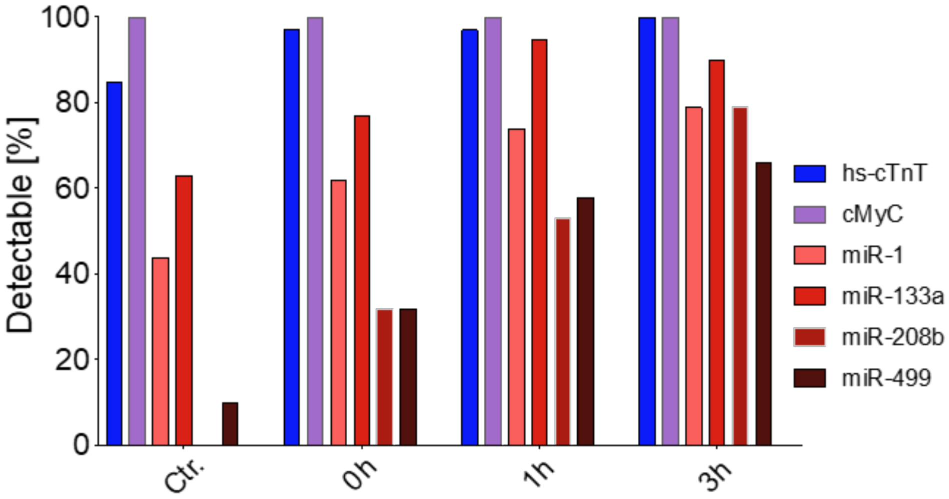

Online Figure XIV. Detectable fraction of cardiac protein and miRNA biomarkers in the acute MI cohort. Shown are percentages of detectable biomarker values in the MI cohort. Values depicted for control patients (Ctr.) and at time point 0 h, 1 h and 3 hrs relative to presentation at hospital. cMyC is best detectable with 100% at all conditions / time points. Muscle-enriched miRNAs miR-1 and miR-133a are better detectable than cardiac-enriched miR-208b and miR-499. This is most strongly pronounced in the control patient group. hs-cTnT = high-sensitive cardiac troponin T; Hs-cTnI = high-sensitive cardiac troponin I; cMyC = cardiac myosin-binding protein C.

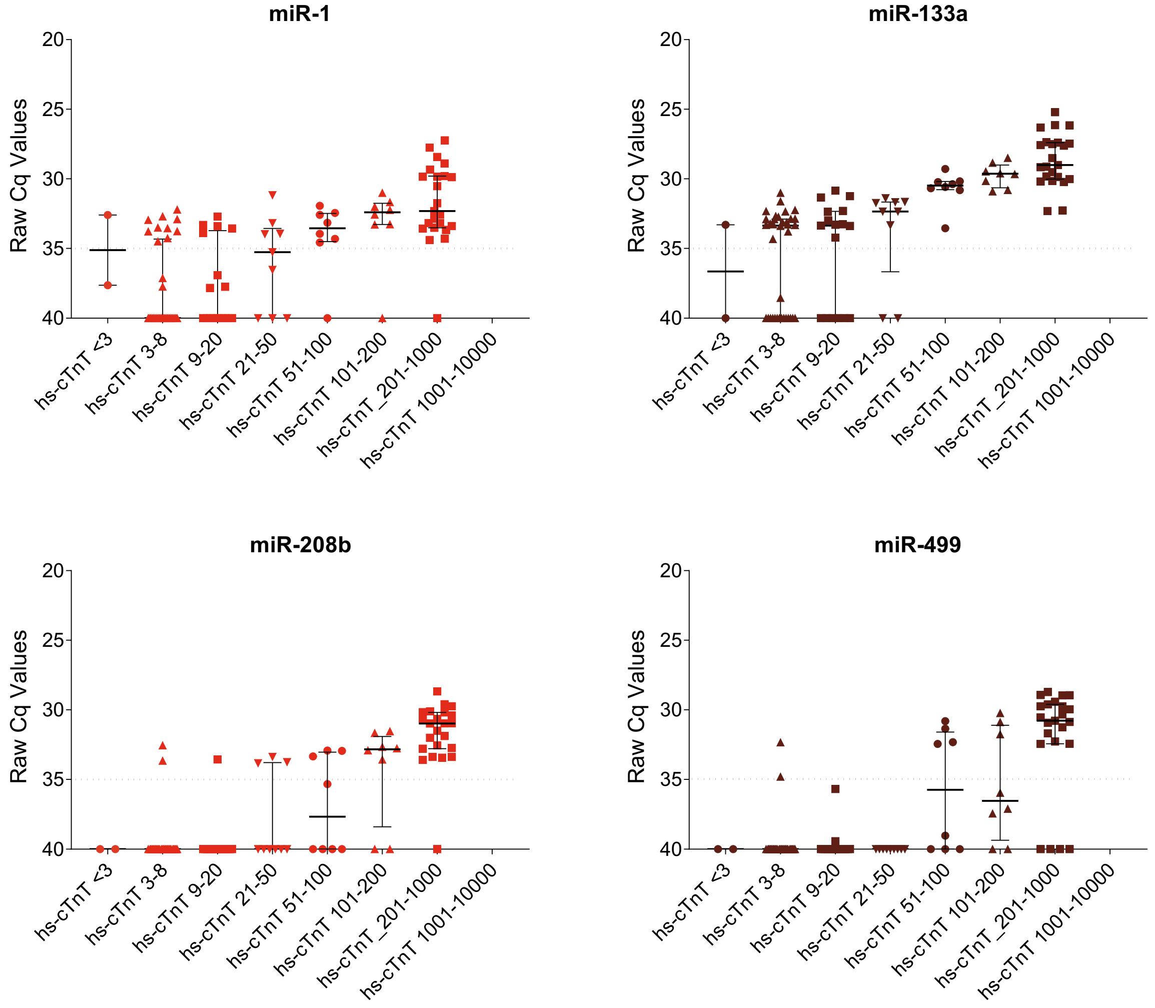

Online Figure XV. miRNA raw expression data corresponding to different ranges of hs-cTnT concentrations (ng/L) with increased RNA input. An additional patient group comprising 57 carefully selected MI samples (n=19 patients at three time points) with hs-cTnT levels of <1,000ng/L was selected and miRNAs quantified. In an attempt to improve detectability, an increased RNA input (2-fold increase) was used. However, detectability remained poor at hs-cTnT levels <50-100ng/L.

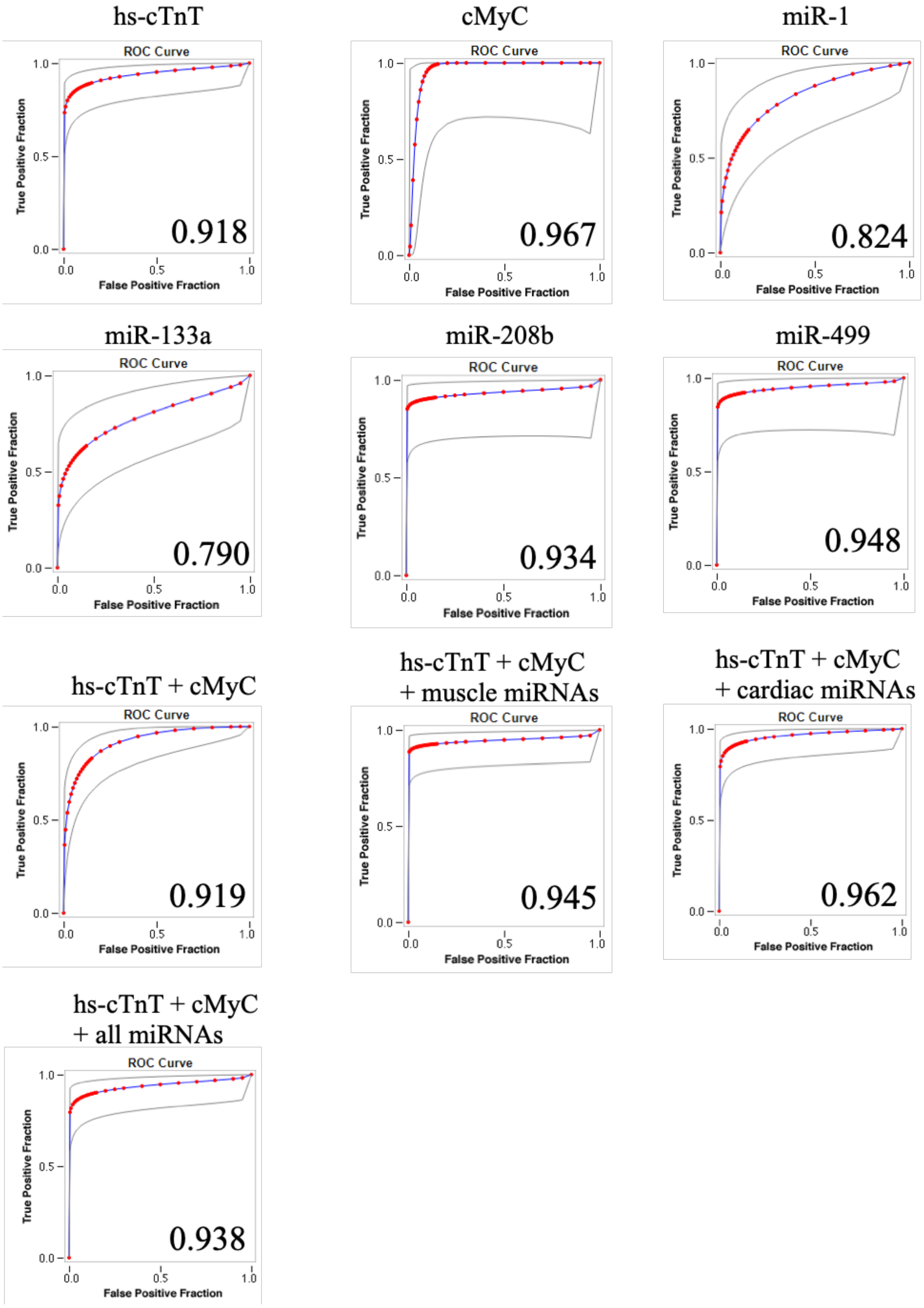

Online Figure XVI. ROC curves for TASH cohort. Grey lines depict the 95% confidence interval of the fitted ROC curve. hs-cTnT, high-sensitive cardiac troponin T; cMyC, cardiac myosin binding protein C.

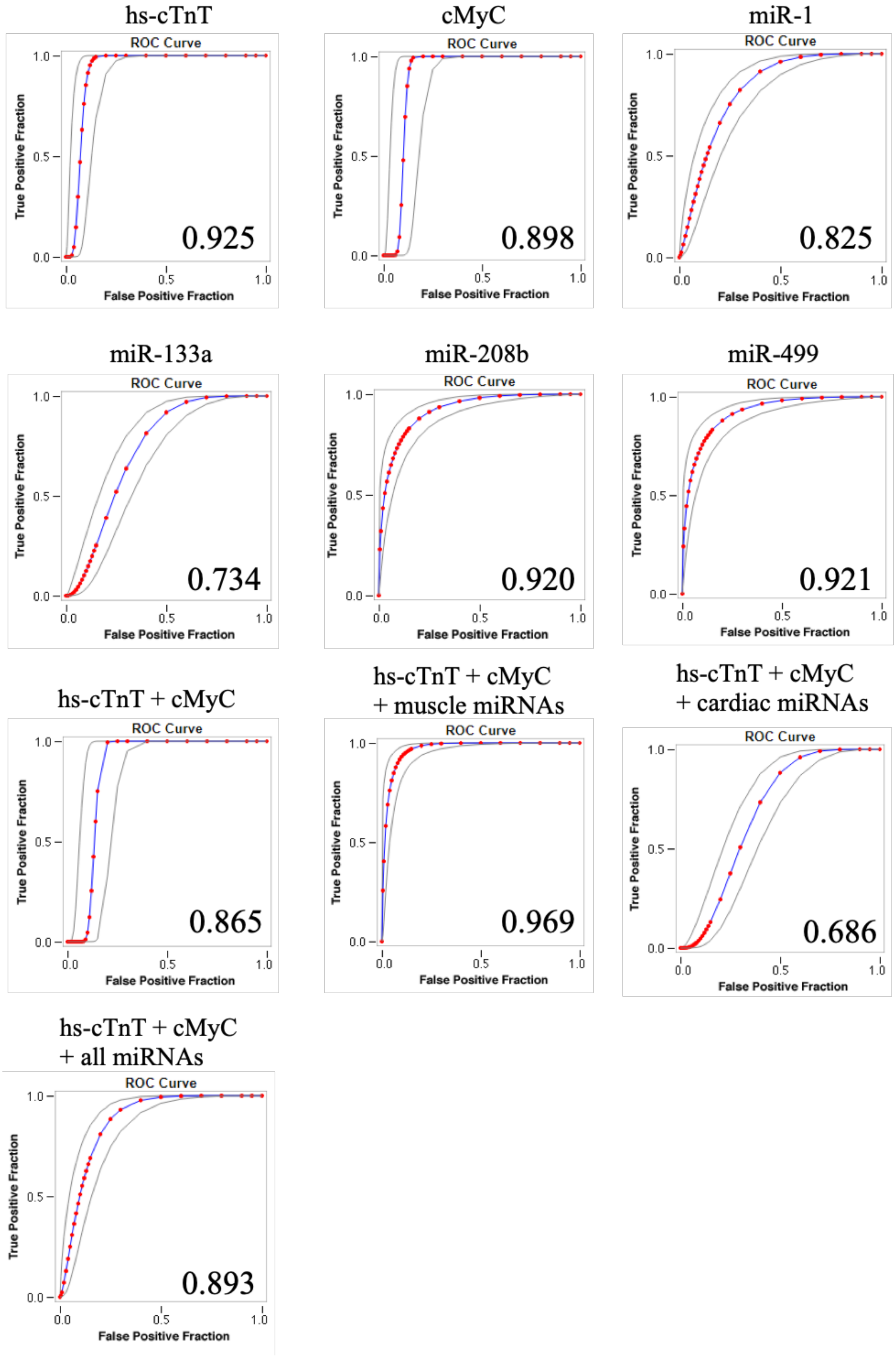

Online Figure XVII. ROC curves for MI cohort. Grey lines depict the 95% confidence interval of the fitted ROC curve. hs-cTnT, high-sensitive cardiac troponin T; cMyC, cardiac myosin binding protein C

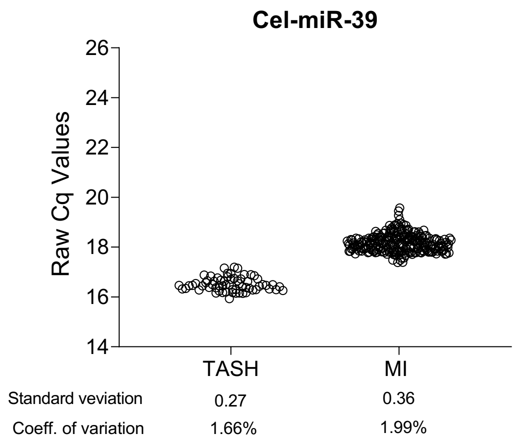

Online Figure XVIII. Raw Cq value distribution of the spike-in normalisation control in the TASH and MI cohort. The distribution of Cel-miR-39 raw Cq values after spike-in in the TASH and MI cohort returned standard deviations < 0.5 and coefficients of variation of <2% indicating good RNA extraction performance and its suitability as normalisation control for relative quantification analysis.

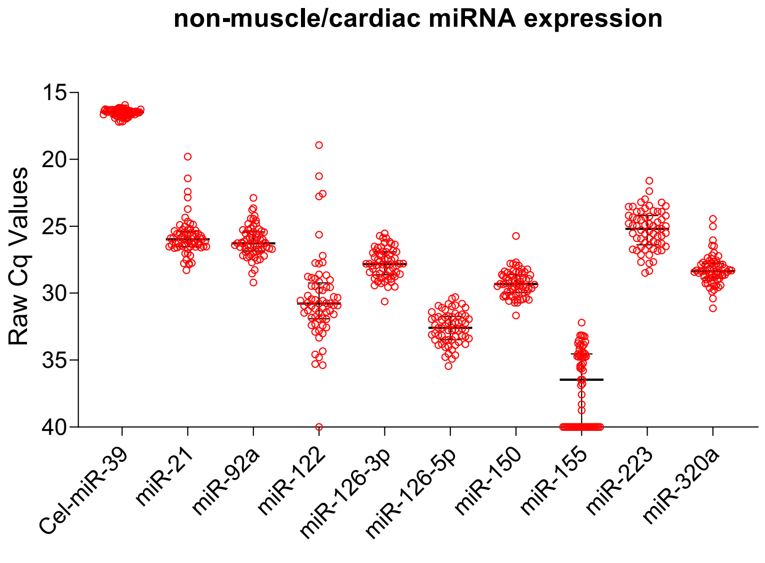

Online Figure XIX. Raw Cq value distribution of non-cardiac/-muscle miRNAs in TASH. Compared to endogenous miRNAs, the Cel-miR-39 spike-in control showed the most stable expression profile across all TASH samples.

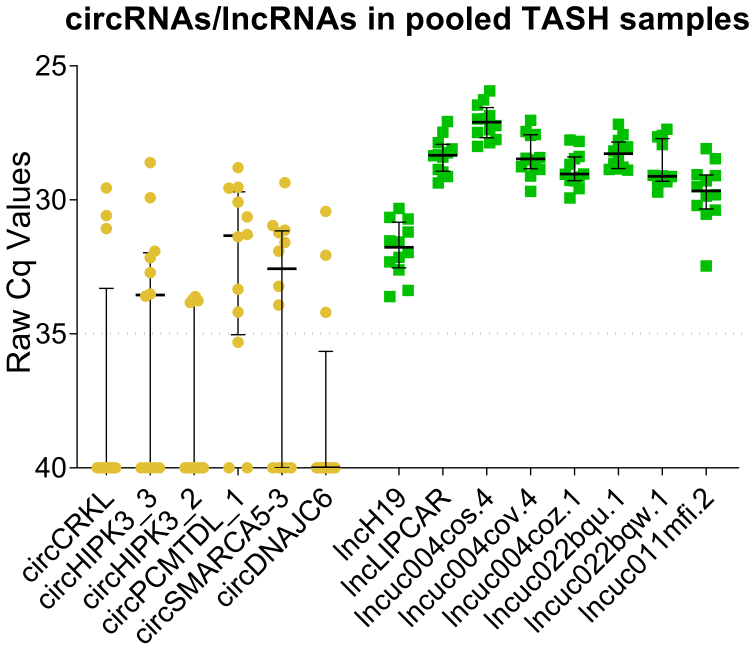

Online Figure XX. Selection of circRNAs and lncRNAs in pooled TASH samples. The figure displays those circRNAs and lncRNAs that were detectable in >50% of 12 pooled TASH samples (4 per time point) or detectable in all samples of one time point (n=3 per time point). The ncRNAs were selected based on detectability (Cq<25 cycles) in cardiac tissue.

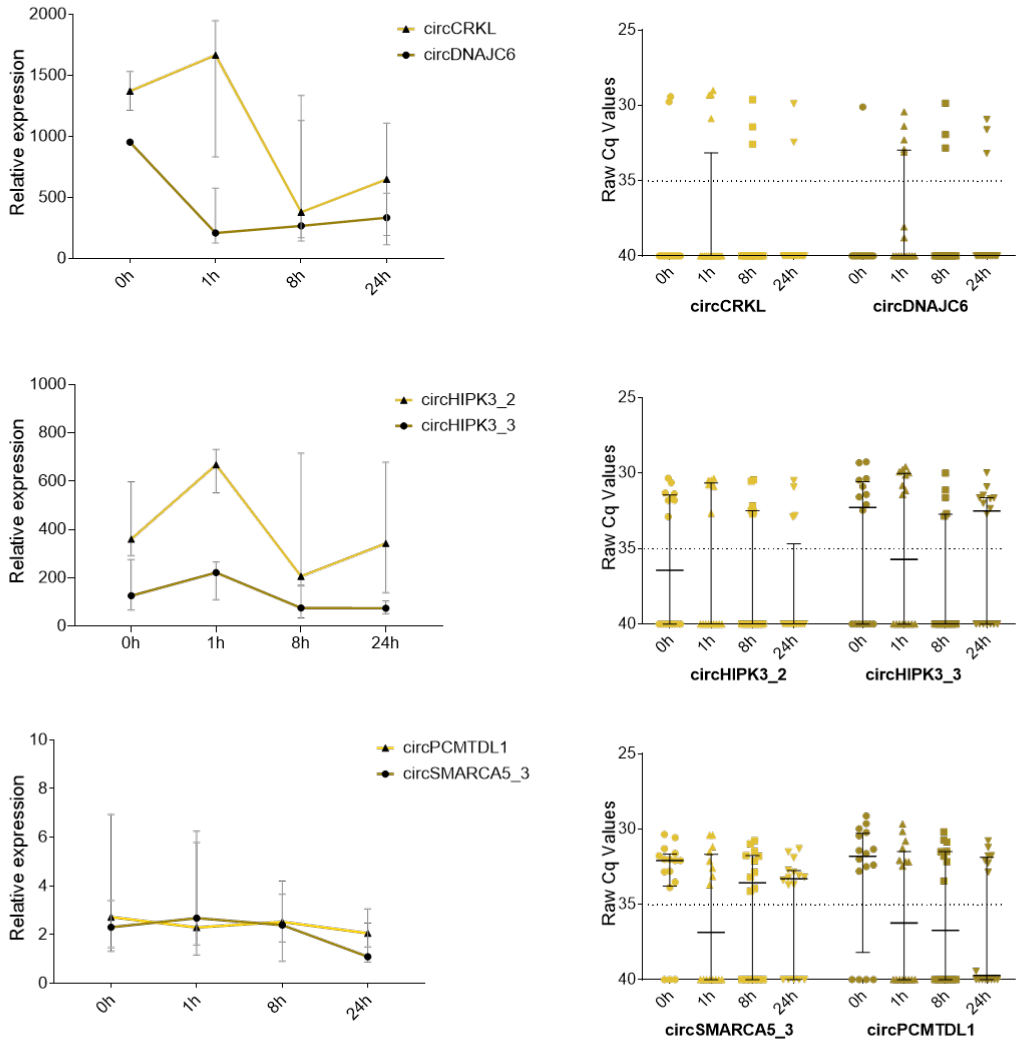

Online Figure XXI. Selected circRNAs tested in the TASH cohort. Results from the evaluation of the selected circRNAs. Only, circSMARCA and circPCMTDL were detectable in >50% per time point, and therefore evaluated in terms of kinetics but showed no dysregulation after myocardial injury.

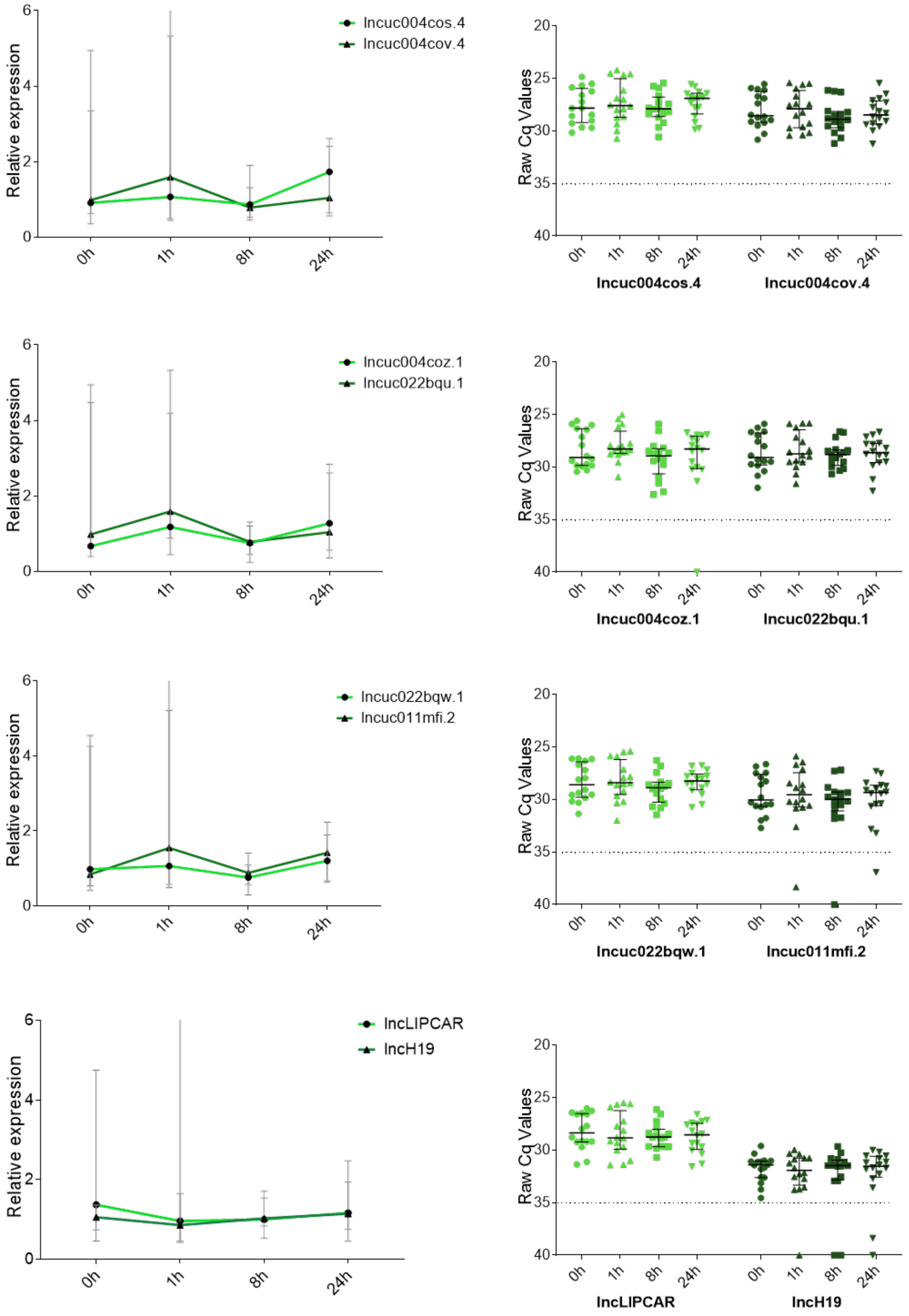

Online Figure XXII. Selected lncRNAs tested in the TASH cohort. Results from the evaluation of the selected circRNAs. All tested lncRNAs were well-detectable at all time points, but showed no dysregulation after onset of myocardial injury (0h).

Online Tables

Online Table I. Primers and Sequences Used for qPCR Detection

ncRNA class	Oligo name	Oligo sequence (5' to 3')
circRNAs		
	circABCD3-5_fwd	TACTTAGACAATGTCCAGTTGGGT
	circABCD3-5_rev	CCTTTTCCTGTTGTGAGACCA
	circAcvr2a_fwd	GATGGCCTACCCTCCTGTACTTG
	circAcvr2a_rev	CCAGGTAGCAAAACAATGCCGCC
	circAdamts6_fwd	GGATGTTCCACTGTTTAAGAGCCAC
	circAdamts6_rev	CCCACCAAAGTGATGGAAACACC
	circALG12_fwd	CTGCGGTTCATCATCTATACCT
	circALG12_rev	CCACTCCTTCCGGCCATACA
	circALPK2-2_fwd	GGCAACACAAATTGCAAGGGA
	circALPK2-2_rev	TCATGTTTCCAACCCCTGTCC
	circAnkib1_fwd	GGCTCCTCTCTTTACTGCTGAAG
	circAnkib1_rev	GATGAGTGCTTTACGGAATTTGGTGG
	circARHGAP32_fwd	GCAAGTTGCAGCGTAATG
	circARHGAP32_rev	TGTCTCATCAGGAACTCCAG
	circAsh1l_fwd	GGTTGGCTATTGGAAGAACAGACCAG
	circAsh1l_rev	CTATCCACAGAGTCATCCCCAGAAG
	circAsph_fwd	CAGATGATCCAGAACAAAAAGCAAAAG
	circAsph_rev	GCCTCCACAGGAACCTGCTCCTC
	circATP2B4_fwd	TCTTCTGCTCTGTAGTCTTGG
	circATP2B4_rev	TTCTTGAATGGCACTGCTTCC
	circATXN10_fwd	GGATGGTATCCCGTTGATCCTGG
	circATXN10_rev	CCTAAAATGAAGGTGCAGGCTCC
	circBach1_fwd	CAGAACAGCTGGATTGTATCC
	circBach1_rev	GTTGTCGGGAAGTTCAGTGG
	circBBS9_fwd	GTCTGGAGTAATGCTAATGAGTTG
	circBBS9_rev	TGCTTGTGGTTGGTAAACAGCT
	circBnc2_fwd	GTTCACTTGAAAGAGATGCACG
	circBnc2_rev	CTGAAGGGTGATGATTTCCTC
	circCACNA1_fwd_2	GTGTGGCCTTAGCAATCTATATTC
	circCACNA1_rev_2	CCTGGAGTAGGGATGTGCTC
	circCACNA1C-12_fwd	TTCCAAAGAGAGGGAGAAGGC
	circCACNA1C-12_rev	AGAACGAAAAAGGATCCAAAGATG
	circCamK2d_fwd	TCTCAGGAGGCACCTCTTTG
	circCamK2d_rev	CGTGCTTTCACATCTTCATCCTC
	circCamsap_fwd	GGCCAGTGTCAAGCGCTTTT
	circCamsap_rev	AGAAGCTTGATAACGGGTGG
	circCamsap1_fwd	CCAGTGTCAAGCGCTTCTCAAC
	circCamsap1_rev	GCTTGGACAGGAGAAGCTTGATAAC
	circCCSER2_fwd	ACTTCAGCTTGCAACTCAGC
	circCCSER2_rev	AGATTCTCTATAGGGAGAGCC
	circCD36-5_fwd	ACTCAGTGTTGGTGTGGTGA

circCD36-5_rev	ACAGGGTACGGAACCAAACTC
circCdr1as_fwd	GTGTCTCCAGTGTATCGGCG
circCdr1as_rev	TACTGGCACCACTGGAAACC
circCDYL_2_fwd	ACAGGCTTAGCTGTTAACGGGA
circCDYL_2_rev	GTCATAGCCTTTCCACCGAACC
circCdyl_fwd	CTGTTCCGGCTCCCAAGTGT
circCDYL_fwd_4	AGGTTTCTGGCCCCGTGACT
circCdyl_rev	AGGCTTAGCTGTTAACGGG
circCDYL_rev_4	CCAGATATTCTGTCTTCCCTTTCTTG
circCHD7_fwd	CAGAAGGCCCAGTGGTAGAAA
circCHD7_rev	GGTGGGGGTGTTTTGTCTAAG
circCRKL_fwd	CTACCTGCAGTTTCCGGTTC
circCRKL_rev	GGTTGGGTGCTGAGACAGAT
circDcbld2_fwd	GGATGTAAGGGTTCCACTCTCAG
circDCBLD2_fwd	TCACAGTGCTGTTCATGAGTG
circDcbld2_rev	CGCGGATTTTTGGCCTCATACTC
circDCBLD2_rev	AGTTGATGGATGTAAGGGTTCC
circDGKD_fwd	GATGAGGTAGACCTGACTGATG
circDGKD_rev	CATAGTAAAGTGTTCGCCCTCG
circDMD-14_fwd	ATTTCACAGGCTGTCACCACC
circDMD-14_rev	TTCGAGGAGGTCTAGGAGGCG
circDMD-18_fwd	GTCCTACAGGATGCTACCCG
circDMD-18_rev	TGACAGCTGTTTGCAGACCT
circDMD-28_fwd	ACTGAAAGAGTTGAATGACTGGC
circDMD-28_rev	GGTGGCCTTGGCAACATTTC
circDMD-39_fwd	GCCAGACCTATTTGACTGGAA
circDMD-39_rev	TGCCAGTGGAGGATTATATTCCAAA
circDMD-41_fwd	GAGGACCCGTGCTTGTAAGT
circDMD-41_rev	CGCAGATTCAGGCTTCCCAA
circDNAJC6_fwd	AACTGCCAAGTTTCACAGCC
circDNAJC6_rev	CATGTCAAAGAGACCTCCCCC
circDNMT3B-4_fwd	TGGAGACTCATTGGAGGACCA
circDNMT3B-4_rev	CAGAGCATGGTACATGGCTTTTC
circELF2_fwd	TTAGAGCAGGGCTATGCTGC
circELF2_rev	TAGAGATGGAGTAGACATCCGG
circERBB2ip_fwd	CATTACCAGCCTCCATTGCA
circERBB2ip_rev	CGACATGGTACCAACCGCAC
circEZH2_fwd	GGATACAGCCTGTGCACATC
circEZH2_rev	TCTGAACCTCTTGAGCTGTC
circFAT1-1_fwd	GAGGACGCCAGAAGAGATGG
circFAT1-1_rev	TGCACGGTGACGTTGTACTC
circFAT1-3_fwd	GAGGCATTTGATCCAGATTCGAG
circFAT1-3_rev	AACTGCAGAGGAGTCTGTTCA
circFBN2-4_fwd	GAATGCCAGGCTATCCCAGG
circFBN2-4_rev	CCACAGGTTGATGATATTTGCCC
circFBXO7-2_fwd	TCTGGTGTTTGGAATGACGA
circFBXO7-2_rev	CCAGTGAGGGGATCCTTGTA
circFGD4-14_fwd	TGAAATGTTGGGAGAAGAAGAAGAC
circFGD4-14_rev	AGAGGTCAAGTCGGTTGACATAA

circFHL2_fwd	GGTGGACAAGCCCTTTGCTG
circFHL2_rev	ATGTACTTCTTGCCGTACAGAG
circFHOD3_fwd	CAGCAGCAACTCTTTCCAATCC
circFHOD3_rev	CCTCCTCTGAATTGGGCTCC
circGIGYF_fwd	AGGGGTGACAGGCGCTTTG
circGIGYF_rev	CTCGGCCATATCGATAATCTGCT
circHERC1-12__rev	GCAACTCCCTCTCTTGTAGCA
circHERC1-12_fwd	GCTGATCGGAGTCAGTGGAG
circHERC2-3_fwd	CTGCCAGTCACAGAATGTGGG
circHERC2-3_rev	TTGACGCCGTCCATGACTGT
circHIPK2-3_fwd	TCGACTTTGGTTCAGCCAGC
circHIPK2-3_rev	ACAGGGGGATGTTCTTGCTC
circHipk3_3_fwd	CGGCCAGTCATGTATCAAAG
circHipk3_3_rev	CCTGGAATACACAACTGCTTGG
circHIPK3_fwd_4	CATGCTGACCTCAAACCAGA
circHIPK3_rev_4	ACACAACCGCTTGGCTCTAC
circHIPK3-2_fwd	TCGGCCAGTCATGTATCAAA
circHIPK3-2_rev	CATTCACATAGGTCCGTGGATA
circKDM5A-6_fwd	AGGAATTTTGGCGGCTGGTA
circKDM5A-6_rev	CTCTCCAAAGCTCTGAAGTGTA
circKIAA1328-2_fwd	TCGAGAGAACTGGTTGCTGA
circKIAA1328-2_rev	TCTGAAGCTGAATCCTGGACTG
circKIF16b_fwd	CAGCTTGTCTATGACCGACC
circKIF16b_rev	TGGCGGCTTCCTTCGGATGG
circLAMA2_fwd	TGACTTCTGTGAACGATGTGC
circLAMA2_rev	TGATTGTAGGCCAAAGGTCC
circLAMA2-34_fwd	TTTTGTGGAGCTCTCCCCTG
circLAMA2-34_rev	GGACCAGTGAGGTTGATGCT
circLRCH3_fwd	TACCTGAACATACAAGCTTGTAAG
circLRCH3_rev	TGGGATATACCGAATACAATTCTGG
circMED13I_fwd_3	GGATATTCTGGTGGGGAGATGA
circMED13I_rev_3	GAAACTTAACAGGATTGGATCATCTT
circMEd13l_2_fwd	CTTAACAGAATTGGATCATCTTGGGC
circMed13l_2_rev	AATTAATCCCTGACTGAATTGGAAAGG
circMef2a_fwd	CTCACAGTGCCAAATGGAGCTGG
circMef2a_rev	GTCCTCCGAGAGTGGACTGTGC
circMETT19_fwd	GCAGGGTCATCCTGGCATT
circMETT19_rev	GAAGATCTGTGTTCCTTGGTC
circMFN2-2_fwd	CATCTCCCAGTCTGCAGTGA
circMFN2-2_rev	CTTTCTGAATCCTGGCGTTG
circMYBPC3-3_fwd	GACTTCAGCTCACTGCTGAAAA
circMYBPC3-3_rev	TCCTTGGTGGACACCTCACA
circMYBPC3-5_fwd	GGCTGAACTTCGACCTGATT
circMYBPC3-5_rev	GCGGCTCCCACTGTACT
circMYH7-16_fwd	AGAAGCGCAAGCTGGAAGAT
circMYH7-16_rev	CCTCCAGCCTCTCGTTCATC
circMYH7-3_fwd	AGACAACCTGGCAGATGCTGAG
circMYH7-3_rev	GGAGCTGCAGGTCATTCTTCTC
circMYH7-7_fwd	GGCACTGATCTCCCAGCTGAC

circMYH7-7_rev	TGTTCATCTCGATCTGCACGGA
circMYL2-1_fwd	GTTCGGGAAATGCTGACCAC
circMYL2-1_rev	GGCAGCAAAGGTGTCTCTCA
circMyl4_fwd	GGGGATGTACTGCGGGCCCTG
circMyl4_rev	GCACTGGCCGTAGGTGATCTTC
circMYL4-1_fwd	TGCGGGGATGTACTGCG
circMYL4-1_rev	GCCGTAGGTGATCTTCATCTCT
circMYOCD_fwd	GAAGATCGCTCTCCGCCCAG
circMYOCD_rev	CTTGGTTAGCCAGCTGCTCC
circMYOCD-1_fwd	CCACTGCAGAGAGGTCCATT
circMYOCD-1_rev	TTAGCCAGTTGTTCCTGGGTC
circMYPN-6_fwd	TCAGAGCCCCACCAATTACT
circMYPN-6_rev	CAGCAGAAGTCGAATCTGTCC
circN4BP2L2_fwd	AAGCACACAAAGACCTCCTC
circN4BP2L2_rev	ACCTGTACCCATCTTGATGG
circNeb_fwd	GTGGACGCCATTCCCCTGTTG
circNeb_rev	GGAGTGCAGCATCTTGGGATCG
circNEBL-23_fwd	AAGTTCTTGATATCCAAAGAGCAAA
circNEBL-23_rev	TTCCATTCCTTTCCCTTTTATC
circNEXN-7_fwd	AAAGGAGTGGCTCTATTCAAGC
circNEXN-7_rev	TGATGCTGATTCAGTTCCCGT
circNFIX_fwd	CTCGAACCCCGGCCTGTGT
circNFIX_rev	GGTTGAACCAGGTGTAGGAGA
circODGH_fwd	GGATGCTGATCTGGACTCCT
circODGH_rev	GCATGGGCCATGGTAGCCAG
circOSBPL10-2_fwd	GCTTCGAGCTTGTGCCAAATA
circOSBPL10-2_rev	CTCCATTAGCAGAGTACACCACC
circPAIP2_fwd	CGAGATCTCCCACAAACTATGG
circPAIP2_rev	CTAGTACTGCTGCGACTTGG
circPCM1-7_fwd	TCGTAGTTCACAACAACCTGT
circPCM1-7_rev	CATCTGTTGCAAAAGGCTCAA
circPCMTD1-1_fwd	CACTTGTCAGCACCTTGCAT
circPCMTD1-1_rev	CTCACAGCTCCTCCCATGAT
circPDLIM5_fwd	AATCCTTGCCCAGATCACTGG
circPDLIM5_rev	TTTTGAACAGGAACCGGCTC
circPDLIM5-16_fwd	TGAAAGAATCTGAAGCCGATAATAC
circPDLIM5-16_rev	CTTTTGAACAGGAACCGGC
circPDLIM5-26_fwd	GTGTTGTGAAAGTTTGGAAGGTCA
circPDLIM5-26_rev	GTCTCACAGTAGGGTTCACCA
circPms1_fwd	GATCTCCTCATGAGCTTTGGTATCC
circPms1_rev	CTTGAAAGGAGTCGAACTGTTGCC
circPPP1R9A-3_fwd	GGTGCTGGTGAGCAGTAACA
circPPP1R9A-3_rev	GTCCAGTTCTGATGGGGAAA
circPRDM5_fwd	TGCACATTCGTACTCACACC
circPRDM5_rev	TTCCTGTAGGCTTGATGCTG
circPtp4a2_fwd	CTCGGTGTCCAGGAGTCTTC
circPtp4a2_rev	GGAGTGACGACTTTGGTTCGAG
circQKI_fwd	GCCCAATTGGGAGCATCTAA
circQKI_rev	CACCGCGTCAGGCAATTCT

circRAPGEF2_fwd	TGTGGATCCTTATCCCATGG
circRAPGEF2_rev	ACAATGCATTCACAGCCACG
circRASA2_fwd	GAGTTAAGACTGAATGAGCTGA
circRASA2_rev	GCGATAAACTTCTTCCTGGTCC
circRBM20-2_fwd	TTAGAGATGGCTTACACAGAAGC
circRBM20-2_rev	GAGACTGGCAGGTGAAGGAAG
circRMST-1_fwd	AAGTAGAGCTCGTGCTGTGAA
circRMST-1_fwd	CCTCAAAGGTATTTGTTTCACCCT
circRNFl69_fwd	CCAGATGATGCAGACCCATC
circRNFl69_rev	CTTTCTCAGGCATCCATACTCC
circRtn4_fwd	CACTCAAGCAGAGATAGAGAGCATAG
circRtn4_rev	GCAGCAGGAAGAGCAAAAAGGGTC
circRtn4bis_fwd	GTCTCTCCAGTACAGGAGGTC
circRtn4bis_rev	GAGGAGTTGGTTCAGAAGTAC
circRunx1_fwd	CTGCCTTTAACCCTCAGCCTCAG
circRunx1_rev	GGCGACTTGCGGTGGGTTTGTG
circRYR2_fwd_1	TCTCTGTATGAGTTGCTGGC
circRYR2_fwd_2	CCTCTCTGCACTGGAGGACAT
circRYR2_fwd_3	TCAAGGACTTTGATGTTGGCG
circRYR2_fwd_4	CATTTACCTTCCGGTCTTCC
circRYR2_rev_1	TTCGATGACCACCACCTTG
circRYR2_rev_2	CCTTGGCTGTCAGTGTGTCAT
circRYR2_rev_3	TTCTCGGCACGGAAGATTCG
circRYR2_rev_4	TCCAGAGAGTCTGCTGGAAAG
circRYR2-113_fwd	AGAGGAAATCGTAAAAACTGTGCT
circRYR2-113_rev	GCCACTATAGGAATGGCGCA
circRYR2-117_fwd	AATCCTCGCCTTGTTCCCTAC
circRYR2-117_rev	TGTCCTCTTGCAAGCCAACATC
circRYR2-124_fwd	TGTCTTTTGCTTGCAATGGT
circRYR2-124_rev	GCCTAGGCACCAGTATTTCA
circSept_fwd	GAGCCTGCCCCTGTGTCTCAGC
circSept_rev	GTGGAGTTGGGTGTCTCGACC
circSLC8A1_2_fwd	GCCGCATGTTGTACATGACACT
circSLC8A1_2_rev	AGAGGTGGAGGGGAGGATTTT
circSlc8a1_fwd	CTGCCAGAGGTGGAGGGGAGG
circSLC8A1_fwd_3	AAAACCATCGAAGGGACTGCC
circSlc8a1_rev	GGTGGGTGAAAGACTTAATCGCCG
circSLC8A1_rev_3	ACACTTCCAACTGTCACAACC
circSLC8A1-1_frev	ATCCCATTGAAAAGGTGGGTGA
circSLC8A1-1_fwd	TGTGAGTGAGAGCATTGGCA
circSlc8aI_fwd_2	TCTGGAGCTCGAGGAAATGT
circSlc8aI_rev_2	TTGGGTGGGAGACTTAATCG
circSMARCA5-3_fwd	TCTCCAAGATGGGCGAAAGT
circSMARCA5-3_rev	TGCACCTCTTTCCAAAATACCAT
circSMARCC1-6_fwd	AAGGAGACTGAAGAGAACAAAGA
circSMARCC1-6_rev	CCAGTCAAGTTCCTCCGACAA
circSORB2_fwd	CGATCGGAGCCAAGGAGTATT
circSORB2_rev	GATAGTAACTCACTGGACTCACG
circSORBS1-7_fwd	GGGAACCCCCAGATAAGAAA

circSORBS1-7_rev	AATCGTTGTGGAGGGATAGG
circSpecc1_fwd	CTGTCTTGCAATGAGCTCAG
circSpecc1_rev	TACTGAAAATTCCCGTGGGG
circSPOPL_fwd	CTCGATCCCCAGTTTTTAATGC
circSPOPL_rev	AGGCAGAGGTGGAGTAGGTT
circSTRN_fwd	ACTTGGAGAACTAGCAGGC
circSTRN_rev	TCGATCTTCACCGCTGTCAG
circSTRN2 2-7_fwd	GATCCAGATACTGAGGAAGCAC
circSTRN2 2-7_rev	TCTTCAAATTCTCTTGACCTTTCC
circSTRN3 2-4_fwd	GCTATTAAGACAGCACGGATTGC
circSTRN3 2-4_rev	TCTTCAAATTCTCTTGACCTTTCC
circTCF25_fwd	GAGGACAGCAGTGGGTTCAG
circTCF25_rev	CGAGGATTCTCCTGTGCAGCT
circTECRL_fwd	TGTCTCATCCCAATCACACAG
circTECRL_rev	TGGCGTAATCTTCTAGCACTC
circTmeff1_fwd	GCTGAGTGTGATGAAGATGCAG
circTmeff1_rev	GGCATGCACATTTCAAACCATCTCC
circTMEM38b_fwd	AGGAGATTGGAAACCAGAAGG
circTMEM38b_rev	AGTGGAGCATAGCAGTAAACC
circTNNI3-2_fwd	GAGACTGGCGCAAGAACATC
circTNNI3-2_rev	CAGAGATCCTCACTCTCCGC
circTNNI3K_fwd	AATGCAAGGTCCTATGCTGC
circTNNI3K_rev	TCTGCTTTGATGGTGTACCG
circTNNT2-6_fwd	AGCGGAAGAGGATGCTGAAG
circTNNT2-6_rev	ATCCGAGGGACAGCTGGG
circTTC3 2-10_fwd	GGCAGTTTAGGACTGTGCAG
circTTC3 2-10_rev	TCCTCATAAGGGTAATCTTCCAG
circTTC3 2-11_fwd	TGCACTTAGCGATGGAAAGAG
circTTC3 2-11_rev	TCCTCATAAGGGTAATCTTCCAG
circTtn_2_fwd	GAAGTGGCCCCTGTTCCTATCC
circTtn_2_rev	CCTTATACCTGTGACTGACACCTCC
circTTN_fwd_1	AAAAGTGGAAGCACCACCAC
circTTN_fwd_2	AAAAGTGGAAGCACCACCAC
circTTN_fwd_3	AGCACAGCCAACCTGAGTCTG
circTTN_fwd_4	CCCAGAGGTGCCAAAGAAAC
circTTN_fwd_5	AGTTGAAGAGGTGGCACCAC
circTTN_rev_1	ATGGATTCTCCCTGCTTTGC
circTTN_rev_2	TGGAGACCCACCGATTTTG
circTTN_rev_3	GACTTTGGGTGTGGCAACTATG
circTTN_rev_4	TGGAGACCCACCGATTTTG
circTTN_rev_5	TGGAGACCCACCGATTTTG
circTTN-275_fwd	GCTAAAGCCAAAGAACAAGAAACT
circTTN-275_rev	TGCGGAAATTACTACCTTTGGT
circTTN-29_fwd	TTATAAAGAAGCCAGTAATTGAAAAA
circTTN-29_rev	TAGGAGGTGGTGGTGCTTTC
circTTN-354_fwd	ACCGGAGGTGCCTAAGAAAA
circTTN-354_rev	CACCGAGTCAACGAATGACA
circTTN-420_fwd	GGTTCTATGTACATTGGAGCAAA
circTTN-420_rev	AGGAGACTGGGGTTAAAAGGA

	circTTN-90_fwd	GGTAGAGAAGGCATTACTACCAAAA
	circTTN-90_rev	TTCTTTGACTTTGGGTGTGG
	circTtn104-110_fwd	AAGAGGGCTACGATGAAGGG
	circTtn104-110_rev	GTACAGTTCCGCTGTGCTTC
	circTtn84-118_fwd	CAAAGAAACCTGCTCCCGAAG
	circTtn84-118_rev	ATCGGTGCTGTTCCAGTGACAT
	circTUSP4_fwd	TCGAGTCACTGTCGCAGAGA
	circTUSP4_rev	TAGACAGAGTCTTCAAATGGCAC
	circTXNDC1_fwd	GCGATGAAGCCGCTTCTCTAC
	circTXNDC1_rev	GGAAGAGGTCAAGGACCGGA
	circUBE2K_fwd	GTTTCTAGTCCAGAATACACCAA
	circUBE2K_rev	GGTGTGTCTGGAGGTCCTGC
	circUGP2_fwd	AAGACCTGGATGGATTTCGG
	circUGP2_rev	TGGAACTGAGAAGCACCATC
	circUTRN_fwd	ATGATGTCTGCCAGAGTTGC
	circUTRN_rev	GGACACTCAGGAGCTGATCAT
	circYYY_fwd	ATGACCTGACATTGCTACTCC
	circYYY_rev	CACAACAAAGGTGCTCCCAG
	circZBTB46-1_fwd	GTTCGAGTACCTGCCCAGAG
	circZBTB46-1_rev	GTTCTTGTGCGCCTTGAAGA
	circZfhx3_fwd	CTACTACACCAACAGCCTGGAGAAG
	circZfhx3_rev	CGAGTCACAGCCTTCCATGGTAAGG
	circZFP148_fwd	GAGCAGATGGACACCCACGA
	circZFP148_rev	TGTGTCAGGTATAAGCCCATCC
	circZfp609_fwd	AAACCGGAGCCAGAGGAAGG
	circZfp609_rev	CAGCTATGTTCTCAGACCTGC
	circZFP827_fwd	CACCTCCAATTCAAAAGATCTGC
	circZFP827_rev	ATGTTCCGGCTGGGTGATAT
	circZNF133-1_fwd	TCATTTTCTAAACCAGAACTCATCA
	circZNF133-1_rev	GGGACCCTGTGGCTTTTT
	circZnf292_2_fwd	CCCAGGAACCATTGGATAAG
	circZnf292_2_rev	CAGAGGTAAGATAAGGTCGG
lncRNAs		
	LIPCAR_fwd	TAAAGGATGCGTAGGGATGG
	LIPCAR_rev	TTCATGATCACGCCCTCATA
	uc004cos.4_fwd	ATGGCCAACCTCCTACTCCT
	uc004cos.4_rev	TAGATGTGGCGGGTTTTAGG
	uc004coz.1_fwd	CAAATCCCTTCTCGTCCCCA
	uc004coz.1_rev	TACCCCCAAGTGTTATGGGC
	uc004cov.4_fwd	TTCCCCAACCTTTTCCTCCG
	uc004cov.4_rev	TGGATAAGTGGCGTTGGCTT
	uc011mfi.2_fwd	ACCGGGGGTATACTACGGTC
	uc011mfi.2_rev	GCTCTAGAGGGGGTAGAGGG
	uc022bqw.1_fwd	TATCCGCCATCCCATACATT
	uc022bqw.1_rev	GGTGATTCCTAGGGGGTTGT
	uc022bqu.1_fwd	GCGGCTTCGACCCTATATCC
	uc022bqu.1_rev	AGGGCTCATGGTAGGGGTAA
	KCNQ10T1_fwd	TTGGTAGGATTTTGTTGAGG
	KCNQ10T1_rev	CAACCTTCCCCTACTACC

MIRT1_fwd	CTTGTTGTCTAAGTGAGTA
MIRT1_rev	TTAATCGTTCCTCTAGTTG
WISPER_fwd	CCATCTGTGGGACATCTGTG
WISPER_rev	TGGGGGCTGTGGAGATAGTA
ENSMUST1611_fwd	CTAAGCGAGGAGCATGGAAC
ENSMUST1611_rev	GCAGCAATTGGCATTGAATA
ENSMUST4047_fwd	CTCTGCATCGACCAGCTTGA
ENSMUST4047_rev	TTCTGGTGTCCTAGGGCATGT
ENST60.2_fwd	TTTTCGGAGGCAGGTTCCAG
ENST60.2_rev	CACCTCGCATGTGCGTTTAT
ENST57.1_fwd	TGGCACTGGGCACTTGATAA
ENST57.1_rev	CAAGTGTGCAGTAGGTATTAGCC
TCONS_701_fwd	GAGGAGCAGTTCATCCCAGTC
TCONS_701_rev	GCAACCACCTGTTCCACGA
RP3.527G5.1_fwd	CCATGTGCTCCTCTCCCTAG
RP3.527G5.1_rev	GGATTAGGGACGTAGGGAGC
RP11.171A24.3_fwd	CGACCCCGGAAAAGTTGTAA
RP11.171A24.3_rev	ACCGGCCCTTTATCTGACAA
RP11.371M22.1_fwd	AAGCAGGCACGGAATTTGAC
RP11.371M22.1_rev	AAATGCGGCCTTGGTGAAAT
H19_fwd	AGAAGGCTGGGGCTCATTTG
H19_rev	GCAGGAGGCATTGCTGATGAT
APOA1-AS_fwd	GCAAACCTTCTTCATCCACCAG
APOA1-AS_rev	GGCACGCTAGTCTGTTGAGTA
THRIL_fwd	TTGGTTGTAGCAGGTCTGGC
THRIL_rev	GGCAACAGAGCAAGACTTCATC
LincRNA-Cox2_fwd	TGGAGGAGTCAGGAGGAATAGG
LincRNA-Cox2_rev	GCTGGCATGGACAAGTTGAAG
LincRNA-p21_fwd	GGGTGGCTCACTCTTCTGGC
LincRNA-p21_rev	TGGCCTTGCCCGGGCTTGTC
SLC26A4-AS1_fwd	TGAAAGGCAGAAGGAAGGGTTT
SLC26A4-AS1_rev	AAGGCAGGTGGATTACGGAAG
HULC_fwd	CAACCTCCAGAACTGTGATCCA
HULC_rev	CTTGCTTGATGCTTTGGTCTGT
NEAT1_fwd	ATGCCACAACGCAGATTGAT
NEAT1_rev	CGAGAAACGCACAAGAAGG
DANCR_fwd	GCCACTATGTAGCGGGTTTC
DANCR_rev	ACCTGCGCTAAGAACTGAGG
lncRMST_fwd	TGAGTGATGGAATAGGTTGCCA
lncRMST_rev	CCTCAAAGGTATTTGTTTCACCCT

Online Table II. Clinical Characteristics of the MI cohort (BACC).

	All (N=83)	Non-AMI (N=45)	AMI (N=38)	p-value
Age (years)	56.0 (48.0, 68.0)	59.0 (48.0, 68.0)	55.0 (48.0, 68.0)	0.99
Male, n (%)	58 (69.9)	29 (64.4)	29 (76.3)	0.35
BMI (kg/m²)	26.3 (24.6, 29.3)	25.5 (23.7, 27.2)	28.5 (25.0, 30.7)	0.019
Systolic Blood Pressure (mmHg)	146.5 (135.4, 161.2)	145.0 (137.7, 155.0)	150.0 (135.0, 168.0)	0.46
Diastolic Blood Pressure (mmHg)	85.5 (78.0, 91.6)	84.0 (77.7, 90.0)	86.0 (78.0, 96.7)	0.33
Hyperlipoproteinemia, n (%)	27 (32.5)	13 (28.9)	14 (36.8)	0.59
Diabetes, n (%)	8 (9.8)	3 (6.7)	5 (13.5)	0.51
Current smoker, n (%)	27 (32.5)	9 (20.0)	18 (47.4)	0.016
History of AMI, n (%)	12 (14.5)	3 (6.7)	9 (23.7)	0.06
History of CAD/Bypass/PCI, n (%)	16 (19.3)	5 (11.1)	11 (28.9)	0.076
Aspirin, n (%)	22 (26.8)	7 (15.6)	15 (40.5)	0.022
Clopidogrel, Prasugrel and/or Ticagrelor, n (%)	7 (8.5)	3 (6.7)	4 (10.8)	0.79

Online Table III. Clinical Characteristics of all MI patients.

	All AMI (N=57)	AMI_discovery (N=38)	AMI_validation (N=19)	p-value
Age (years)	62.0 (49.7, 69.0)	55.0 (48.0, 68.0)	67.0 (59.2, 70.7)	0.13
Male, n (%)	46 (80.7)	29 (76.3)	17 (89.5)	0.41
BMI (kg/m²)	27.5 (24.8, 30.4)	28.5 (25.0, 30.7)	27.1 (24.7, 28.8)	0.4
Systolic Blood Pressure (mmHg)	150.0 (138.7, 169.0)	150.0 (135.0, 168.0)	152.5 (141.0, 174.5)	0.52
Diastolic Blood Pressure (mmHg)	88.0 (79.3, 98.7)	86.0 (78.0, 96.7)	96.0 (81.8, 105.1)	0.096
Hyperlipoproteinemia, n (%)	21 (36.8)	14 (36.8)	7 (36.8)	1
Diabetes, n (%)	8 (14.3)	5 (13.5)	3 (15.8)	1
Current smoker, n (%)	28 (49.1)	18 (47.4)	10 (52.6)	0.93
History of AMI, n (%)	13 (22.8)	9 (23.7)	4 (21.1)	1
History of CAD/Bypass/PCI, n (%)	18 (31.6)	11 (28.9)	7 (36.8)	0.76
Aspirin, n (%)	22 (39.3)	15 (40.5)	7 (36.8)	1
Clopidogrel, Prasugrel and/or Ticagrelor, n (%)	6 (10.7)	4 (10.8)	2 (10.5)	1

Online Table IV. ROC Analysis in TASH cohort.

Biomarker	AUC
**hs-cTnT**	0.918
**cMyC**	0.967
**Tnl-PEA**	0.912
**miR-1**	0.824
**miR-133a**	0.79
**miR-208b**	0.934
**miR-499**	0.948
**hs-cTnT + cMyC**	0.919
**hs-cTnT+TnI-PEA**	0.918
**hs-cTnT+miR-1**	0.869
**hs-cTnT+miR-133a**	0.888
**hs-cTnT+miR-208b**	0.943
**hs-cTnT+miR-499**	0.957
**miR-1+miR-133**	0.874
**miR-208+miR-499**	0.952
**hs-cTnT+miR-1+miR-133a**	0.904
**hs-cTnT+miR-208b+miR-499**	0.939
**hs-cTnT+cMyC+miR-1+miR133a**	0.945
**hs-cTnT+cMyC+miR-208b+miR499**	0.962
**hs-cTnT+cMyC+miR-1+miR133a+miR-208b+miR-499**	0.938
**hs-cTnT+Tnl-PEA+miR-1+miR133a**	0.848
**hs-cTnT+Tnl-PEA+miR-208b+miR499**	0.936
**hs-cTnT+Tnl-PEA+cMyC+miR-1+miR133a**	0.941
**hs-cTnT+Tnl-PEA+cMyC+miR-208b+miR499**	0.9
**hs-cTnT+Tnl-PEA+miR-1+miR-133a+miR-208b+miR-499**	0.886
**hs-cTnT+Tnl-PEA+cMyC+miR-1+miR-133a+miR-208b+miR-499**	0.942

Green font = best-performing biomarker;
Red font = worst-performing biomarker.
AUC, area under the curve;
cMyC, cardiac myosin-binding protein C;
hs-cTnT, high-sensitive cardiac troponin T.

Online Table V. ROC Analysis in the AMI cohort.

Biomarker	AUC
**hs-cTnT**	0,925
**cMyC**	0.898
**TnI-PEA**	0,695
**miR-1**	0,825
**miR-133a**	0,734
**miR-208b**	0,92
**miR-499**	0,921
**hs-cTnT + cMyC**	0.865
**hs-cTnT + cMyC + miR-1 + miR-133**	0.969
**hs-cTnT + cMyC + miR-208 + miR-499**	0,686
**hs-cTnT + cMyC + miR-1 + miR133a + miR-208b + miR-499**	0.893
**hs-cTnT + TnI-PEA + cMyC + miR-1 + miR-133**	0,828
**hs-cTnT + TnI-PEA + cMyC + miR-208 + miR-499**	0,917
**hs-cTnT + cMyC + TnI-PEA + miR-1 + miR133a + miR-208b + miR-499**	0,852

Green font = best-performing biomarker;
Red font = worst-performing biomarker;
AUC, area under the curve;
cMyC, cardiac myosin-binding protein C;
hs-cTnT, high-sensitive cardiac troponin T.

Online Table VI. Previous Publications on miRNAs in MI or Mycoardial Injury.

Publication	miRNAs	Cq cutoff	Heparinase	Low hs-cTnT measures
**Ai et al. Biochem Biophys Res Commun 2009**	miR-1	30	no heparinase	no
**Wang et al. Eur Heart J 2010**	miR-1, miR-133a, miR-208a, miR-499	40	no heparinase	no
**D'Alessandra et al. Eur Heart J 2010**	miR-1, miR-133a, miR-133b, miR-499	40	no heparinase	no
**Adachi et al. Clin Chem 2010**	miR-499	no (40)	no heparinase	no
**Cheng et al. Clin Sci 2013**	miR-1	no (40)	no heparinse	no
**Kuwabara et al. Circ Cardiovasc Genet 2011**	miR-1, miR-133a	40	no heparinase	no
**Long et al. Int J Biol Sci 2012**	miR-1, miR-126	40	no heparinase	no
**Oerlemans et al. EMBO Mol Med 2012**	miR-1, miR-21, miR-146a, miR-208a, miR-499	no (40)	no heparinase	yes
**Devaux et al. Clin Chem 2012**	miR-208b, miR-499	no (40)	no heparinase	no
**Corsten et al. Circ Cardiovasc Genet 2012**	miR-208b, miR-499	no (40)	no heparinase	no
**Olivieri et al. Int J Cardiol 2013**	miR-499	35	before heparin adminstration	yes
**Vogel et al. Clin Chem 2013**	numerous not previously described	no (40)	no heparinase	yes
**Liebetrau et al. JACC 2013**	miR-1, miR-133a, miR-208a	no (40)	no heparinase	no

hs-cTnT, high-sensitive cardiac troponin T.
