# Supplementary material for: Comparative Analysis of Circulating Noncoding RNAs Versus Protein Biomarkers in the Detection of Myocardial Injury
Source: Circ Res. 2019 Jun 4;125(3):328–40. doi: 10.1161/CIRCRESAHA.119.314937 (PMC6641471; doi:10.1161/CIRCRESAHA.119.314937)
Supplement: Supplementary file 2 [file res-125-328-s002.pdf]

Gemma Bridges-Lyman

From: Ojeda Echevarria, Francisco Miguel <f.ojeda-echevarria@uke.de>
Sent: Monday, May 20, 2019 7:04 AM
To: circres
Cc: christian.schulte@kcl.ac.uk
Subject: CIRCRES/2019/314937R1 - acknowledgement

*** **CAUTION:** This email originated from outside of the **American Heart Association**. Do **not** click links or open attachments unless you recognize the sender and know the content is safe. ***

Dear Sir or Madam,
I would like to express my agreement regarding the acknowledgement of the manuscript CIRCRES/2019/314937R1: A Comparative Analysis of Circulating Non-Coding RNAs versus Protein Biomarkers in the Detection of Myocardial Injury

Best regards,
Francisco M. Ojeda

Francisco M. Ojeda, PhD
Statistiker, leitender Statistiker

Universitäres Herz- und Gefäßzentrum Hamburg
Klinik für Allgemeine und Interventionelle Kardiologie

Universitätsklinikum Hamburg-Eppendorf
Christoph-Probst-Weg 1-2
3 OG, Raum 03.1.06.1
20246 Hamburg
Telefon +49 (0)40 7410-28287
f.ojeda-echevarria@uke.de
www.uhz.de

Universitätsklinikum Hamburg-Eppendorf; Körperschaft des öffentlichen Rechts; Gerichtsstand: Hamburg | www.uke.de
Vorstandsmitglieder: Prof. Dr. Burkhard Göke (Vorsitzender), Prof. Dr. Dr. Uwe Koch-Gromus, Joachim Prölß, Marya Verdel

SAVE PAPER - THINK BEFORE PRINTING
