# Supplementary material for: Comparative Analysis of Circulating Noncoding RNAs Versus Protein Biomarkers in the Detection of Myocardial Injury
Source: Circ Res. 2019 Jun 4;125(3):328–40. doi: 10.1161/CIRCRESAHA.119.314937 (PMC6641471; doi:10.1161/CIRCRESAHA.119.314937)

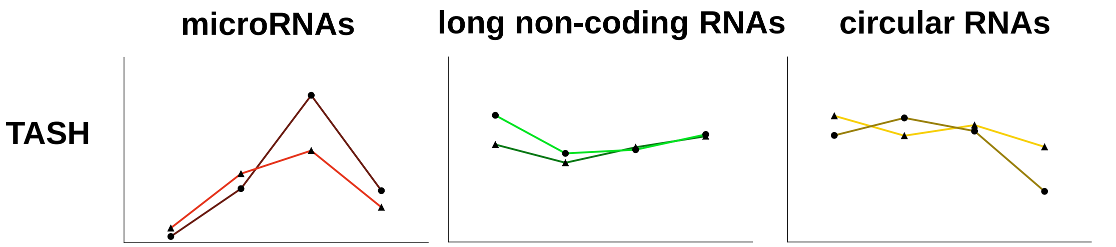

		Cardiac					Muscle		
		hs-cTnI	cMyC	CK-MB	miR-208b	miR-499	CK	miR-1	miR-133a
STEMI	hs-cTnT	0.96	0.92	0.93	0.87	0.93	0.88	0.75	0.84
NSTEMI	hs-cTnT	0.96	0.93	0.88	0.81	0.88	0.77	0.72	0.75

Depicted are regression coefficients; p for all combinations <0.0001

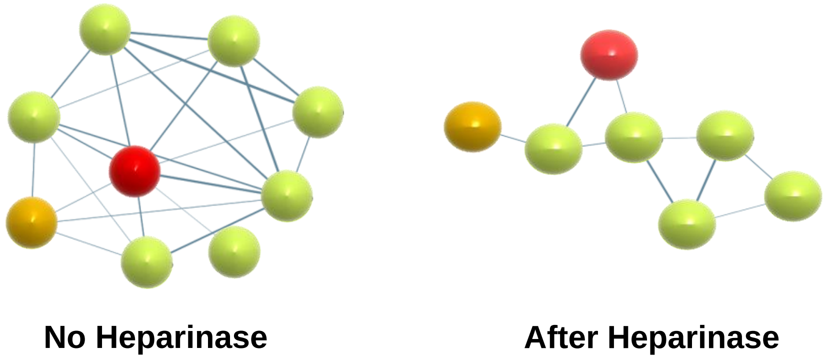

Supplement: Supplementary file 3 [file res-125-328-s003.pdf]
